# Recurrent Hospitalization Among Patients With Atrial Fibrillation Undergoing Intracoronary Stenting Treated With 2 Treatment Strategies of Rivaroxaban or a Dose-Adjusted Oral Vitamin K Antagonist Treatment Strategy

**DOI:** 10.1161/CIRCULATIONAHA.116.025783

**Published:** 2017-01-23

**Authors:** C. Michael Gibson, Duane S. Pinto, Gerald Chi, Douglas Arbetter, Megan Yee, Roxana Mehran, Christoph Bode, Jonathan Halperin, Freek W.A. Verheugt, Peter Wildgoose, Paul Burton, Martin van Eickels, Serge Korjian, Yazan Daaboul, Purva Jain, Gregory Y.H. Lip, Marc Cohen, Eric D. Peterson, Keith A.A. Fox

**Affiliations:** From Cardiovascular Division, Department of Medicine, Beth Israel Deaconess Medical Center, Harvard Medical School, Boston, MA (C.M.G., D.S.P., G.C., D.A., M.Y., S.K., Y.D., P.J.); Cardiovascular Institute, Mount Sinai Medical Center, Icahn School of Medicine at Mount Sinai, New York (R.M., J.H.); Heart Center, Department for Cardiology and Angiology I, University of Freiburg, Freiburg, Germany (C.B.); Onze Lieve Vrouwe Gasthuis, Amsterdam, the Netherlands (F.W.A.V.); Janssen Pharmaceuticals, Inc, Beerse, Belgium (P.W., P.B.); Bayer Pharmaceuticals, Inc, Berlin, Germany (M.v.E.); University of Birmingham Centre for Cardiovascular Sciences, City Hospital, Birmingham, United Kingdom (G.Y.H.L.); Division of Cardiology, Newark Beth Israel Medical Center, Newark, NJ (M.C.); Duke Clinical Research Institute, Durham, NC (E.D.P.); and Centre for Cardiovascular Science, University of Edinburgh and Royal Infirmary of Edinburgh, Edinburgh, United Kingdom (K.A.A.F.).

**Keywords:** atrial fibrillation, percutaneous coronary intervention, rivaroxaban, vitamin K

## Abstract

**Background:**

Patients with atrial fibrillation who undergo intracoronary stenting traditionally are treated with a vitamin K antagonist (VKA) plus dual antiplatelet therapy (DAPT), yet this treatment leads to high risks of bleeding. We hypothesized that a regimen of rivaroxaban plus a P2Y_12_ inhibitor monotherapy or rivaroxaban plus DAPT could reduce bleeding and thereby have a favorable impact on all-cause mortality and the need for rehospitalization.

**Methods:**

Stented subjects with nonvalvular atrial fibrillation (n=2124) were randomized 1:1:1 to administration of reduced-dose rivaroxaban 15 mg daily plus a P2Y_12_ inhibitor for 12 months (group 1); rivaroxaban 2.5 mg twice daily with stratification to a prespecified duration of DAPT of 1, 6, or 12 months (group 2); or the reference arm of dose-adjusted VKA daily with a similar DAPT stratification (group 3). The present post hoc analysis assessed the end point of all-cause mortality or recurrent hospitalization for an adverse event, which was further classified as the result of bleeding, a cardiovascular cause, or another cause blinded to treatment assignment.

**Results:**

The risk of all-cause mortality or recurrent hospitalization was 34.9% in group 1 (hazard ratio=0.79; 95% confidence interval, 0.66–0.94; *P*=0.008 versus group 3; number needed to treat=15), 31.9% in group 2 (hazard ratio=0.75; 95% confidence interval, 0.62–0.90; *P*=0.002 versus group 3; number needed to treat=10), and 41.9% in group 3 (VKA+DAPT). Both all-cause death plus hospitalization potentially resulting from bleeding (group 1=8.6% [*P*=0.032 versus group 3], group 2=8.0% [*P*=0.012 versus group 3], and group 3=12.4%) and all-cause death plus rehospitalization potentially resulting from a cardiovascular cause (group 1=21.4% [*P*=0.001 versus group 3], group 2=21.7% [*P*=0.011 versus group 3], and group 3=29.3%) were reduced in the rivaroxaban arms compared with the VKA arm, but other forms of rehospitalization were not.

**Conclusions:**

Among patients with atrial fibrillation undergoing intracoronary stenting, administration of either rivaroxaban 15 mg daily plus P2Y_12_ inhibitor monotherapy or 2.5 mg rivaroxaban twice daily plus DAPT was associated with a reduced risk of all-cause mortality or recurrent hospitalization for adverse events compared with standard-of-care VKA plus DAPT.

**Clinical Trial Registration:**

URL: http://www.clinicaltrials.gov. Unique identifier: NCT01830543.

## Abstract 2

Supplemental Digital Content is available in the text.

**Editorial, see p 334**

Approximately 5% to 8% of patients undergoing coronary stent implantation also have atrial fibrillation (AF).^1–3^ Among patients undergoing first-generation stent implantation, dual antiplatelet therapy (DAPT) is superior to vitamin K antagonist (VKA),^4^ but among patients with AF, VKA is superior to DAPT in reducing the risk of ischemic stroke.^5^ As a result, a common practice has been to combine DAPT and VKA to manage patients who have both a stent and AF.^6^ Unfortunately, this strategy, often referred to as triple therapy, has been associated with major bleeding rates of 4% to 12% over the course of the first year of treatment.^7^

The primary results of the PIONEER study (An Open-label, Randomized, Controlled, Multicenter Study Exploring Two Treatment Strategies of Rivaroxaban and a Dose-Adjusted Oral Vitamin K Antagonist Treatment Strategy in Subjects With Atrial Fibrillation Who Undergo Percutaneous Coronary Intervention) demonstrated that among subjects with AF undergoing intracoronary stent placement, administration of either rivaroxaban 15 mg daily plus P2Y_12_ monotherapy for 1 year or rivaroxaban 2.5 mg twice daily plus 1, 6, or 12 months of DAPT at physician discretion significantly reduced the risk of clinically significant bleeding (TIMI [Thrombolysis in Myocardial Infarction] major+TIMI minor+bleeding requiring medical attention) compared with the current standard-of-care VKA plus 1, 6, or 12 months of DAPT with comparable efficacy, although the confidence intervals (CIs) were broad for efficacy.^8^ We hypothesized that a significant reduction in bleeding with favorable trends in overall efficacy would also be associated with a significant reduction in all-cause mortality or recurrent hospitalization.

## Methods

### Study Oversight

The executive committee, in conjunction with the sponsor, designed the study. All statistical analyses were performed by the PERFUSE (Perfusion Use in Stroke Evaluation Study) group using a copy of the Study Data Tabulation Model database. The academic members of the executive committee drafted the manuscript and made all revisions. Both national and institutional regulatory agencies and ethics committees approved the study. An independent data and safety monitoring board monitored the scientific integrity and the safety of the trial.

### Study Population

Details of the trial design have been published previously.^9^ In brief, the trial enrolled men and women >18 years of age with paroxysmal, persistent, or permanent nonvalvular AF who underwent percutaneous coronary intervention with stent placement. Major exclusion criteria included clinically significant bleeding within the past 12 months, a creatinine clearance <30 mL/min, anemia of unknown cause with a hemoglobin <10 g/dL, significant gastrointestinal bleeding within the past 12 months or any condition known to increase the risk of bleeding, a prior stroke or transient ischemic attack, stent placement during the index hospitalization for in-stent restenosis, and stent thrombosis during the index hospitalization. All subjects provided written informed consent.

### Study Protocol and Treatment Strategies

Subjects were randomized within 72 hours of sheath removal once the international normalized ratio was ≤ 2.5. The responsible clinician prespecified the intended duration of DAPT (1, 6, or 12 months) and the intended P2Y_12_ inhibitor (clopidogrel, prasugrel, or ticagrelor) before randomization. Subjects were randomized in a 1:1:1 fashion to rivaroxaban 15 mg (or 10 mg for subjects with moderate renal impairment [creatinine clearance 30–50 mL/min]) once daily plus background single antiplatelet therapy with clopidogrel 75 mg once daily (or ticagrelor 90 mg twice daily or prasugrel 10 mg once daily in up to 15% of subjects per group) for 12 months (although aspirin could be administered up to 24 hours before the first dose of study drug, it was to be withheld after randomization; group 1); rivaroxaban 2.5 mg twice daily plus background DAPT with low-dose aspirin (75–100 mg/d) plus clopidogrel 75 mg once daily (or ticagrelor 90 mg twice daily or prasugrel 10 mg once daily in up to 15% of subjects per group) for a prespecified duration of either 12 months or for 1 or 6 months followed by rivaroxaban 15 mg (or 10 mg for subjects with moderate renal impairment) once daily plus background single antiplatelet therapy with low-dose aspirin (75–100 mg; group 2); or traditional triple therapy with dose-adjusted VKA once daily to achieve a target international normalized ratio of 2.0 to 3.0 plus background DAPT with low-dose aspirin (75–100 mg/d) plus clopidogrel 75 mg once daily (or ticagrelor 90 mg twice daily or prasugrel 10 mg once daily in up to 15% of subjects per group) for a prespecified duration of either 12 months or for 1 or 6 months followed by dose-adjusted VKA once daily (target international normalized ratio, 2.0–3.0) plus background single antiplatelet therapy with low-dose aspirin (75–100 mg; group 3).

### End Points

The primary end point of the substudy was the occurrence of all-cause death or rehospitalization for an adverse event. Adverse events were defined according to the International Conference on Harmonization guidelines. Study investigators were responsible for reporting all adverse events and indicating the seriousness of the event, as well as whether the event resulted in inpatient hospitalization. Those adverse events that resulted in hospitalization are included in this analysis. Two physicians (C.M.G. and G.C.) blinded to study drug assignment were provided with a list of adverse event terms associated with rehospitalization. All adverse events were classified as potentially attributable to bleeding, cardiovascular causes, or other causes through consensus. All adverse event terms, the number of events per term, and their categorization are shown in Table I in the online-only Data Supplement).

### Statistical Analysis

SAS version 9.4 was used to perform all statistical analyses. All patients who received at least 1 dose of the study drug were included in the analysis; subjects were analyzed on an as-treated basis; and for the primary analysis, the data were pooled across all strata of DAPT duration (1, 6, 12 months) as prespecified. The cumulative percentages of all deaths and rehospitalizations for an adverse event observed from the time of the first study drug was first administered up to 2 days after discontinuation of the study drug were calculated. Two specific pairwise comparisons were made simultaneously (group 1 versus 3 and group 2 versus 3) with no adjustment to the type I error rate of 0.05. A Cox proportional hazard model was used to compare the time from administration of the first dose of study drug to the first occurrence any cause of death or hospitalization for an adverse event with treatment group as a covariate to provide a point estimate (hazard ratio [HR]) and 2-sided 95% CI. Cumulative event rates were summarized at 360 days with the Kaplan-Meier method. The Wei-Lin-Weissfeld method was used to calculate unadjusted HRs and 95% CIs for the multiple event analysis.^10^ The Wei-Lin-Weissfeld method uses a semiparametric marginal Cox distribution and takes into account all multiple events of interest that a subject has during the study versus a traditional time to first event analysis. Because of the nonindependent nature of these data, sandwich variance estimation was used. Six subjects from 1 site (n=4 in the rivaroxaban groups versus n=2 in the VKA group) were excluded from all analyses because of violations of Good Clinical Practice guidelines before unblinding. A value of *P*<0.05 was considered statistically significant. This study was a nonprespecified post hoc analysis.

## Results

From May 10, 2013, through July 30, 2015, a total of 2124 subjects were randomized. The baseline characteristics of the subjects were well matched, as reported in the primary publication^8^ (Table 1 and Table II in the online-only Data Supplement). The median age was 71 years (interquartile range, 64–77 years), and 25.6% of subjects were women. The median CHADS_2_, CHA_2_DS_2_-VASc, and HAS-BLED scores were 2, 4, and 3, respectively.^8^ The time in therapeutic range for the internationalized normalized ratio was 65% and did not vary by region.^8^ No patients were lost to follow-up, and the ascertainment of all-cause death was 100% complete in this trial.^8^

**Table 1.**
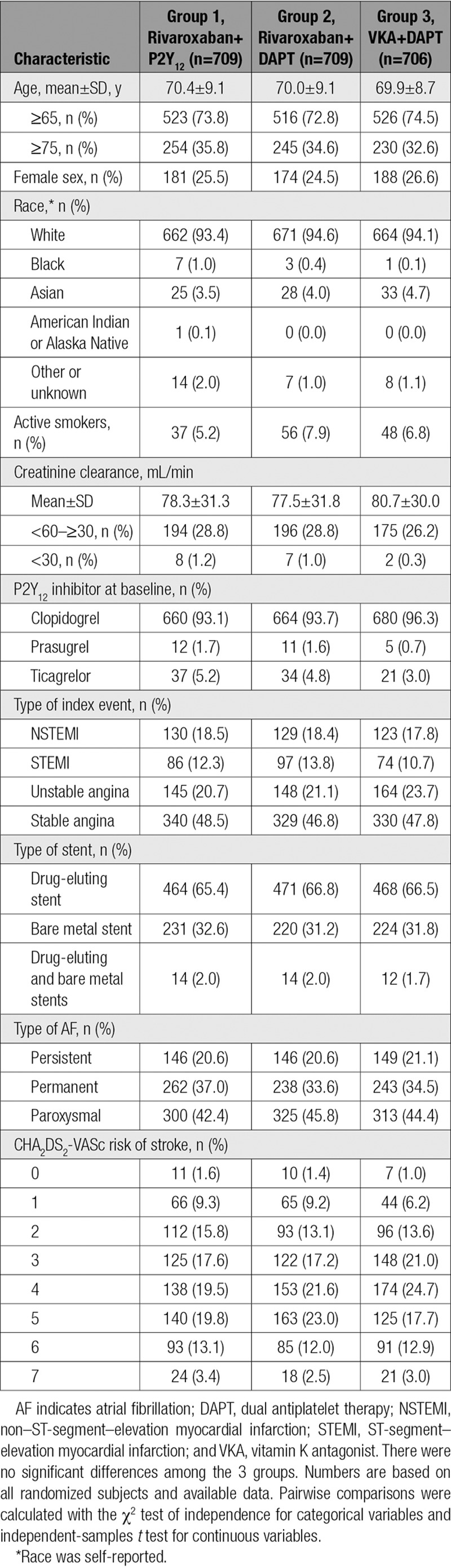
Baseline Characteristics

**Figure 1.**
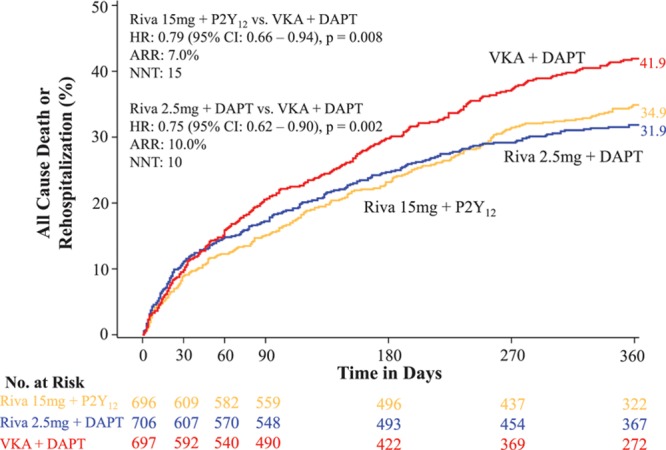
**Time to all-cause death or first recurrent hospitalization.** The treatment-emergent period is the period starting after the first study drug administration following randomization and ending 2 days after the study drug was stopped. Hazard ratios (HRs) compared with the vitamin K antagonist (VKA) group are based on the Cox proportional hazards model. Rehospitalizations do not include first index event hospitalization. Log-rank *P* values compared with the VKA group are based on the 2-sided log-rank test. ARR indicates absolute risk reduction; NNT, number needed to treat; Riva+DAPT, rivaroxaban 2.4 mg twice daily plus background dual antiplatelet therapy with low-dose aspirin; and Riva+P2Y_12_, rivaroxaban 15 mg once daily+P2Y_12_ inhibitor.

The risk of all-cause mortality or recurrent hospitalization was 34.9% in group 1 (rivaroxaban 15 mg once daily+P2Y_12_ inhibitor; HR= 0.79; 95% CI, 0.66–0.94; *P*=0.008 versus group 3 [VKA+DAPT]; number needed to treat=15), 31.9% in group 2 (rivaroxaban 2.5 mg twice daily+DAPT; HR=0.75; 95% CI, 0.62–0.90; *P*=0.002 versus group 3 [VKA+DAPT]; number needed to treat=10), and 41.9% in group 3 (reference group of VKA+DAPT; Table 2 and Figure 1). No significant interaction terms were found in subgroup analyses (Figures I and II in the online-only Data Supplement), including duration of DAPT. Both all-cause death plus hospitalization potentially for bleeding (group 1=8.6% [*P*=0.032 versus group 3], group 2=8.0% [*P*=0.012 versus group 3], and group 3=12.4%) and all-cause death plus rehospitalization potentially for a cardiovascular cause (group 1=21.4% [*P*=0.001 versus group 3], group 2=21.7% [*P*=0.011 versus group 3], and group 3=29.3%) were reduced in the rivaroxaban arms compared with the VKA arm. No reductions were seen for either rivaroxaban arm compared with the VKA arm for other causes of rehospitalization or for all-cause death (Table 2).

**Table 2.**
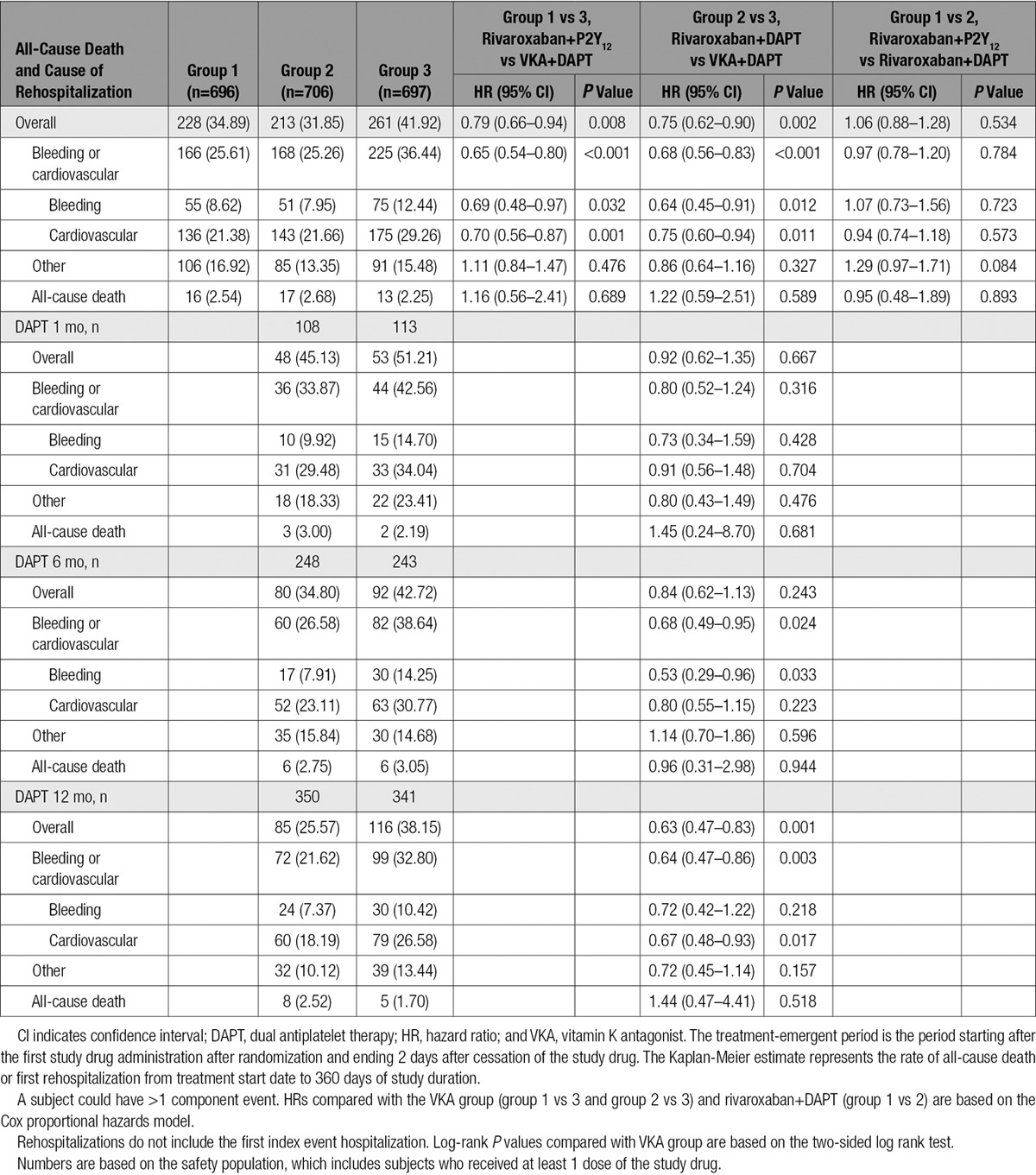
Kaplan-Meier Estimates and HRs for All-Cause Death or First Recurrent Hospitalization

**Figure 2.**
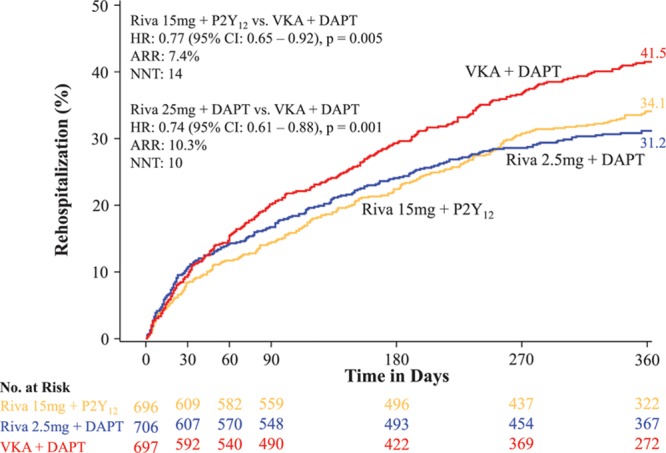
**Time to first recurrent hospitalization.** The treatment-emergent period is the period starting after the first study drug administration following randomization and ending 2 days after the study drug was stopped. Hazard ratios (HRs) compared with the vitamin K antagonist (VKA) group are based on the Cox proportional hazards model. Rehospitalizations do not include first index event hospitalization. Log-rank *P* values compared with VKA group are based on the 2-sided log-rank test. ARR indicates absolute risk reduction; NNT, number needed to treat; Riva+DAPT, rivaroxaban 2.4 mg twice daily plus background dual antiplatelet therapy with low-dose aspirin; and Riva+P2Y_12_, rivaroxaban 15 mg once daily+P2Y_12_ inhibitor.

**Figure 3.**
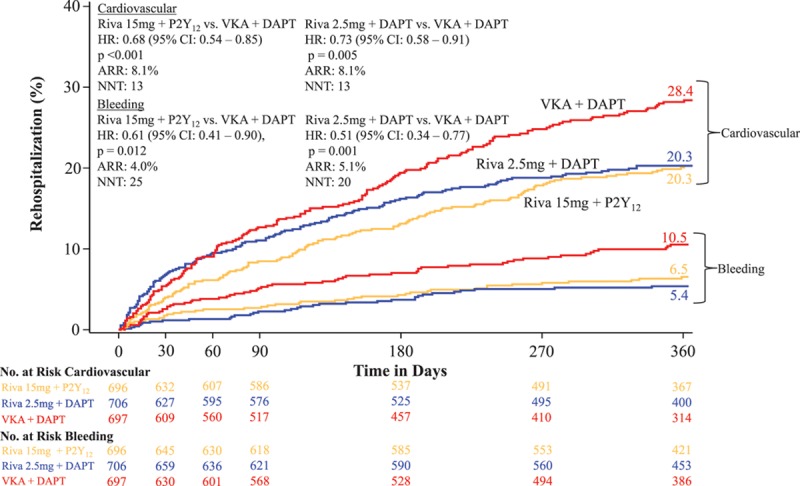
**Time to first recurrent hospitalization caused by cardiovascular or bleeding event.** The treatment-emergent period is the period starting after the first study drug administration following randomization and ending 2 days after the study drug was stopped. Hazard ratios (HRs) compared with the vitamin K antagonist (VKA) group are based on the Cox proportional hazards model. Rehospitalizations do not include first index event hospitalization. Log-rank *P* values compared with the VKA group are based on the 2-sided log-rank test. ARR indicates absolute risk reduction; NNT, number needed to treat; Riva+DAPT, rivaroxaban 2.4 mg twice daily plus background dual antiplatelet therapy with low-dose aspirin; and Riva+P2Y_12_, rivaroxaban 15 mg once daily+P2Y_12_ inhibitor.

**Figure 4.**
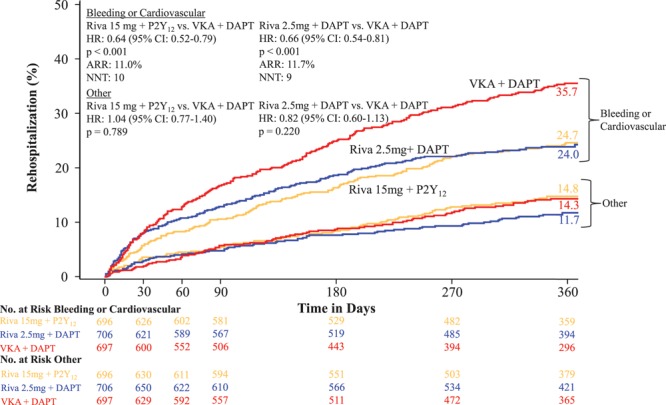
**Time to first recurrent hospitalization caused by combined bleeding or cardiovascular event or other event.** The treatment-emergent period is the period starting after the first study drug administration following randomization and ending 2 days after the study drug was stopped. Hazard ratios (HRs) compared with the vitamin K antagonist (VKA) group are based on the Cox proportional hazards model. Rehospitalizations do not include first index event hospitalization. Log-rank *P* values compared with the VKA group are based on the 2-sided log-rank test. ARR indicates absolute risk reduction; NNT, number needed to treat; Riva+DAPT, rivaroxaban 2.4 mg twice daily plus background dual antiplatelet therapy with low-dose aspirin; and Riva+P2Y_12_, rivaroxaban 15 mg once daily+P2Y_12_ inhibitor.

The rate of all-cause rehospitalization was 34.1% in group 1 (rivaroxaban 15 mg once daily+P2Y_12_ inhibitor; HR=0.77; 95% CI, 0.65–0.92; *P*=0.005 versus group 3 [VKA+DAPT]), 31.2% in group 2 (rivaroxaban 2.5 twice daily+DAPT; HR=0.74; 95% CI, 0.61–0.88; *P*=0.001 versus group 3 [VKA+ DAPT]), and 41.5% in group 3 (reference arm of VKA+DAPT; Table 3 and Figure 2). The relative reduction in recurrent hospitalization was greater for bleeding events, but the absolute reduction in recurrent hospitalization was greater for cardiovascular events (Table 3 and Figure 3). There were no significant interaction terms in the assessment of subgroups, including DAPT duration (Figures III and IV in the online-only Data Supplement). Although the above analysis assessed the time to the first event, some patients were hospitalized on >1 occasion. The risk of multiple rehospitalizations for any given subject showed a magnitude of event reduction similar to that observed for the time to first event reduction (group 1 versus group 3: HR=0.78, 95% CI, 0.64–0.96 *P*=0.005; group 2 versus group 3: HR=0.75, 95% CI, 0.61–0.92, *P*=0.001; Table 4). There was a highly significant reduction in bleeding or cardiovascular end points combined, but rehospitalization for other causes were not reduced (Table 3 and Figure 4). All adverse events resulting in hospitalization were classified as severe, moderate, or mild. Significant reductions were seen in moderate adverse events, the most common classification, for both rivaroxaban arms. Adverse events categorized as severe bleeding events were reduced in the 15 mg rivaroxaban plus P2Y_12_ monotherapy arm (*P*=0.021) and the 2.5 mg rivaroxaban plus DAPT arm (*P*=0.003), and trends favoring the rivaroxaban arms were seen for a reduction in severe and mild events in general (Table III in the online-only Data Supplement).

**Table 3.**
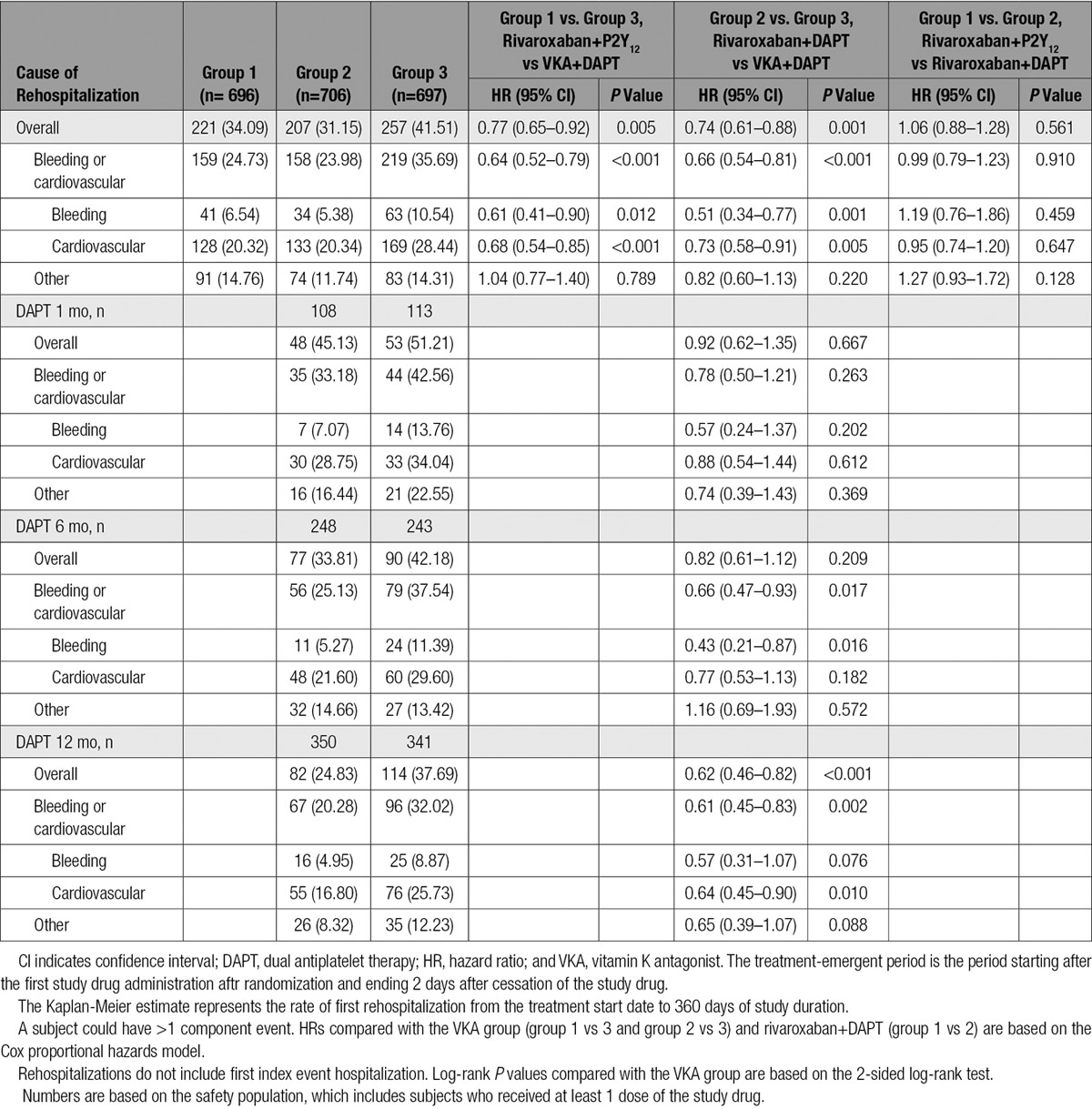
Kaplan-Meier Estimates and HRs for First Recurrent Hospitalization

**Table 4.**
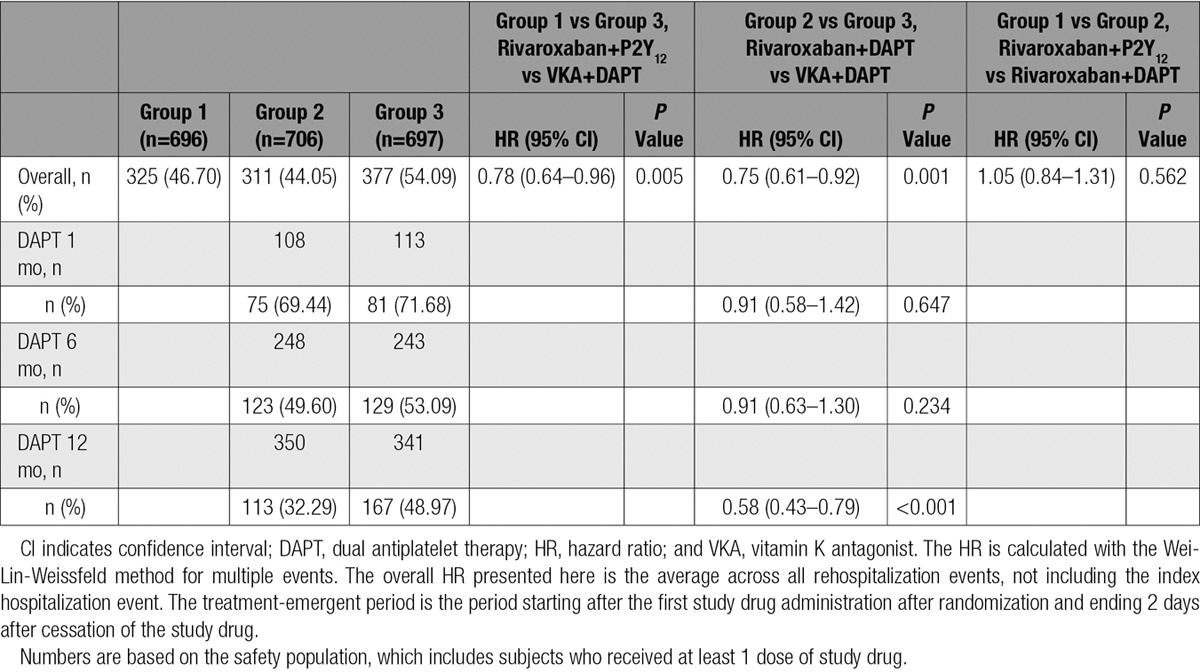
HRs and 95% CIs for Time to Multiple Recurrent Hospitalizations

## Discussion

Among patients with AF undergoing coronary stent placement, the administration of rivaroxaban in either of 2 dose strategies was associated with a reduced risk of all-cause mortality or recurrent hospitalization for any adverse event compared with a VKA plus DAPT. There was a reduction in the risk of both a first rehospitalization and all (any) rehospitalizations for adverse events. The absolute reduction in cardiovascular events was greater but the relative reduction was greater for bleeding in the rivaroxaban arms. The number needed to treat with rivaroxaban to prevent 1 death or hospitalization ranged from 10 for 2.5 mg rivaroxaban+DAPT to 15 for 15 mg rivaroxaban+P2Y_12_ inhibitor. The results of the present analysis add to and strengthen the primary results of the study and demonstrate that the reduction in bleeding and efficacy events was clinically meaningful insofar as it often resulted in fewer hospitalizations in these patients. Although the results of this analysis demonstrate a statistically significant improvement in clinical events, there is also the potential to improve healthcare value because rehospitalization may be costly.

Both the present analysis and the primary report^8^ of the primary safety end point (TIMI major+TIMI minor+bleeding requiring medical attention) demonstrated a reduction in bleeding events. In contrast to the results presented here, however, there was no difference in the prespecified occurrence of the rigorously adjudicated composite secondary end point of death, myocardial infarction, and stroke.^8^ Recurrent hospitalization is a more frequent end point and is ascertained with greater sensitivity but less specificity than the traditional adjudicated end point of death/myocardial infarction and stroke. As a result, the present analysis had much greater statistical power (90%) to ascertain a 20% difference in the treatment strategies (the magnitude observed in the present analysis), whereas the end points of cardiovascular death/myocardial infarction and stroke had only 16.8% power to ascertain a 20% treatment difference (Table IV in the online-only Data Supplement). Although many hospitalizations did not qualify as a death/myocardial infarction or stroke, underlying thrombosis or ischemia still may have played a role in the hospitalization. The fact that bleeding and cardiovascular events differed among the strategies but other causes of hospitalization did not supports the acceptable specificity of rehospitalization as an end point.

Although adjudication of events in clinical trials is often based on rigorous definitions and meticulously collected source documents, it still relies on an adjudication process, which, although conducted by experts, may still be somewhat subjective. Prior studies have demonstrated that conclusions related to the adjudication of clinical end points often vary across adjudicators, clinical sites, and core laboratories dedicated to an end point, for instance.^11^ The rate of concordance varies significantly according to the experience and judgment of the adjudicator, the availability and quality of source documents, and the type of the end point itself.^11^ Advantages of using all-cause mortality or rehospitalization as end points are the near certainty of the occurrence and robust documentation of the events (eg, death certificate or insurance claims data or trial data documenting hospital admission), making them objective end points that do not require an adjudicator’s interpretation. As a result of these potential advantages, these end points have been referred to as the gold standard of clinical events.^12^ In contrast, identification of cause-specific mortality or nonfatal events such as myocardial infarction or stroke may be complex, inconsistent, and often inferred (eg, assuming that all unidentified causes of death are cardiovascular deaths). Although subjects with myocardial infarctions and strokes are hospitalized to establish these diagnoses, deaths may occur without hospitalization, and for this reason, it is critical that all-cause death be added to the end point of hospitalization. In addition, all-cause mortality and rehospitalization are comprehensive end points that encompass the occurrence of both efficacy and safety events. For example, in evaluations of all-cause mortality, death resulting from a myocardial infarction (efficacy end point) carries a similar weight as death resulting from significant gastrointestinal bleeding (safety end point) with no need to distinguish between two. Prior studies such as the ATHENA trial (A Trial With Dronedarone to Prevent Hospitalization or Death in Patients With Atrial Fibrillation), which supported the approval of dronedarone for treatment of nonpermanent AF, have used the composite of hospitalization and all-cause death as a primary means to evaluate the efficacy and safety of a therapeutic strategy.^13,14^

Hospitalizations may not have been due to a death/myocardial infarction or stroke but may nonetheless be associated with a poor quality of life and higher costs. Because costs for rehospitalizations after percutaneous coronary intervention involving bleeding and cardiovascular events are substantial, a 10% absolute and a 25% relative reduction in the risk of hospitalization would likely be associated with a reduction in healthcare costs. The costs of a bleeding event associated with a VKA is estimated to be approximately US $8000 (2011 estimate),^15,16^ similar to costs for common cardiovascular conditions such as chest pain (≈$8000), heart failure (≈$10 000), and percutaneous coronary intervention (≈$25 000) that result in rehospitalization. In addition, the total cost of international normalized ratio monitoring per year has been estimated to be $2134 in the first year and $1170 per year thereafter as long as stable levels have been attained.^17^

The results of the study stratified by DAPT duration are of interest to practicing clinicians. However, there are limitations to exploring the results in these subgroups. There was a negative interaction term for DAPT duration for the end point of all-cause mortality and rehospitalization, and the validity of interrogating these subgroups is questionable. The decision to treat patients with 1, 6, or 12 months of DAPT was not randomized and was based on clinician preference. As might be expected, there were imbalances in patient characteristics across the DAPT duration strata (Table V in the online-only Data Supplement) and imbalances in patient characteristics within each DAPT duration stratum across the 3 treatment strategies (Tables VI–VIII in the online-only Data Supplement). In addition to imbalances in these identified confounders, there are likely imbalances in unidentified confounders. Last, there was no adjustment for multiplicity in testing for these subgroups and others.

The results presented here are generally applicable to those patients treated with clopidogrel. Given the small number of patients treated with novel thienopyridines, additional trials would be required to more rigorously assess both the safety and efficacy of concomitant therapy with prasugrel or ticagrelor in a larger population.

### Limitations

The present analysis is a post hoc analysis. No adjustment was made to account for multiple testing. Accordingly, statistically significant differences between the groups should be interpreted in this context. The method of allocating events to bleeding, cardiovascular, or other causes was not described a priori. This methodology could be prospectively applied by others using the extensive tables provided in the online-only Data Supplement that describe how these adverse events can be mapped into the 3 categories. This analysis is based on a randomized, controlled trial with specific inclusion and exclusion criteria (including the exclusion of patients at high risk of bleeding), and the results of the study may not be generalizable to all patients in clinical practice. Hospital bills and length of stay were not collected to assess costs. Although the 2.5 mg twice daily plus DAPT dosing regimen is currently indicated and available in Europe and a number of other countries for the secondary prevention of acute coronary syndrome events, the 15/10 mg once daily dosing strategy studied here is currently not approved for the management of patients with either acute coronary syndrome or AF. Sites were unblinded with respect to warfarin therapy, although clinical event categorization was blinded. It could be argued that there was a general bias to admit more patients on open-label VKA to the hospital. The increase in rehospitalization, however, was attributable exclusively to bleeding and cardiovascular causes alone. There was no difference among the 3 strategies with respect to rehospitalization for all other types of adverse events, indicating that clinicians were not biased in attributing all types of additional hospitalizations in general to VKA+DAPT.

## Sources of Funding

This study was funded by the sponsors, Janssen Scientific Affairs, LLC, and Bayer.

## Disclosures

All authors have received research grant support from Janssen Scientific Affairs, LLC, and Bayer, the sponsors of the study. Drs Burton and Wildgoose are employees of Janssen, a Johnson & Johnson affiliate. Dr Eickels is an employee of Bayer AG. Dr Gibson has received consulting fees from Janssen Scientific Affairs, LLC, and Bayer. Drs Lip and Halperin have received consulting fees from Janssen. Dr Cohen is part of the Janssen speakers bureau and has received research grant support and advisory board honoraria.

## Supplementary Material

## Clinical Perspective

### What Is New?

- Coronary stent patients with atrial fibrillation who are managed with triple therapy (vitamin K antagonist+dual antiplatelet therapy) face a high risk of bleeding.
- This substudy of the PIONEER trial (An Open-label, Randomized, Controlled, Multicenter Study Exploring Two Treatment Strategies of Rivaroxaban and a Dose-Adjusted Oral Vitamin K Antagonist Treatment Strategy in Subjects With Atrial Fibrillation Who Undergo Percutaneous Coronary Intervention) demonstrates that either rivaroxaban 15 mg daily plus P2Y_12_ inhibitor monotherapy or rivaroxaban 2.5 mg twice daily plus dual antiplatelet therapy is associated with a reduced risk of all-cause mortality or recurrent hospitalization for adverse events compared with standard-of-care vitamin K antagonist plus dual antiplatelet therapy.

### What Are the Clinical Implications?

- Rehospitalizations potentially attributable to either bleeding or cardiovascular events were reduced with both rivaroxaban strategies.

## References

[1] Rubboli A, Colletta M, Herzfeld J, Sangiorgio P, Di Pasquale G. (2007). Periprocedural and medium-term antithrombotic strategies in patients with an indication for long-term anticoagulation undergoing coronary angiography and intervention.. Coron Artery Dis.

[2] Wang TY, Robinson LA, Ou F-S, Roe MT, Ohman EM, Gibler WB, Smith SC, Peterson ED, Becker RC. (2008). Discharge antithrombotic strategies among patients with acute coronary syndrome previously on warfarin anticoagulation: physician practice in the CRUSADE registry.. Am Heart J.

[3] Pérez-Gómez F, Alegría E, Berjón J, Iriarte JA, Zumalde J, Salvador A, Mataix L, NASPEAF Investigators (2004). Comparative effects of antiplatelet, anticoagulant, or combined therapy in patients with valvular and nonvalvular atrial fibrillation: a randomized multicenter study.. J Am Coll Cardiol.

[4] Leon MB, Baim DS, Popma JJ, Gordon PC, Cutlip DE, Ho KK, Giambartolomei A, Diver DJ, Lasorda DM, Williams DO, Pocock SJ, Kuntz RE. (1998). A clinical trial comparing three antithrombotic-drug regimens after coronary-artery stenting: Stent Anticoagulation Restenosis Study Investigators.. N Engl J Med.

[5] Connolly S, Pogue J, Hart R, Pfeffer M, Hohnloser S, Chrolavicius S, Pfeffer M, Hohnloser S, Yusuf S. (2006). Clopidogrel plus aspirin versus oral anticoagulation for atrial fibrillation in the Atrial fibrillation Clopidogrel Trial with Irbesartan for prevention of Vascular Events (ACTIVE W): a randomised controlled trial.. Lancet.

[6] Camm AJ, Kirchhof P, Lip GY, Schotten U, Savelieva I, Ernst S, Van Gelder IC, Al-Attar N, Hindricks G, Prendergast B, Heidbuchel H, Alfieri O, Angelini A, Atar D, Colonna P, De Caterina R, De Sutter J, Goette A, Gorenek B, Heldal M, Hohloser SH, Kolh P, Le Heuzey JY, Ponikowski P, Rutten FH. (2010). Guidelines for the management of atrial fibrillation: the Task Force for the Management of Atrial Fibrillation of the European Society of Cardiology (ESC).. Europace.

[7] Paikin JS, Wright DS, Crowther MA, Mehta SR, Eikelboom JW. (2010). Triple antithrombotic therapy in patients with atrial fibrillation and coronary artery stents.. Circulation.

[8] Gibson CM, Mehran R, Bode C, Halperin J, Verheugt F, Wildgoose P, Van Eickels M, Lip GY, Cohen M, Husted S, Peterson E, Fox K. (2015). An open-label, randomized, controlled, multicenter study exploring two treatment strategies of rivaroxaban and a dose-adjusted oral vitamin k antagonist treatment strategy in subjects with atrial fibrillation who undergo percutaneous coronary intervention (PIONEER AF-PCI).. Am Heart J.

[9] Gibson CM, Mehran R, Bode C, Halperin J, Verheugt F, Wildgoose P, Birmingham M, Ianus J, Burton P, van Eickels M, Korjian S, Daaboul Y, Szlosek D, Lip GY, Cohen M, Husted S, Peterson E, Fox K. (2016). An open-label, randomized, controlled, multicenter study exploring two treatment strategies of rivaroxaban and a dose-adjusted oral vitamin K antagonist treatment strategy in subjects with atrial fibrillation who undergo percutaneous coronary intervention: PIONEER AF-PCI.. N Engl J Med.

[10] Wei LJ, Lin D.Y., Weissfeld L. (1989). Regression analysis of multivariate incomplete failure time data by modeling marginal distributions.. J Am Stat Assoc.

[11] Popma CJ, Sheng S, Korjian S, Daaboul Y, Chi G, Tricoci P, Huang Z, Moliterno DJ, White HD, Van de Werf F, Harrington RA, Wallentin L, Held C, Armstrong PW, Aylward PE, Strony J, Mahaffey KW, Gibson CM. (2016). Lack of concordance between local investigators, angiographic core laboratory, and clinical event committee in the assessment of stent thrombosis: results from the TRACER Angiographic Substudy.. Circ Cardiovasc Interv.

[12] Allen LA, Spertus JA. (2013). End points for comparative effectiveness research in heart failure.. Heart Fail Clin.

[13] Hohnloser SH, Crijns HJ, van Eickels M, Gaudin C, Page RL, Torp-Pedersen C, Connolly SJ, ATHENA Investigators (2009). Effect of dronedarone on cardiovascular events in atrial fibrillation.. N Engl J Med.

[14] Torp-Pedersen C, Crijns HJ, Gaudin C, Page RL, Connolly SJ, Hohnloser SH, ATHENA Investigators (2011). Impact of dronedarone on hospitalization burden in patients with atrial fibrillation: results from the ATHENA study.. Europace.

[15] Kim MM, Metlay J, Cohen A, Feldman H, Hennessy S, Kimmel S, Strom B, Doshi JA. (2010). Hospitalization costs associated with warfarin-related bleeding events among older community-dwelling adults.. Pharmacoepidemiol Drug Saf.

[16] Ghate SR, Biskupiak J, Ye X, Kwong WJ, Brixner DI. (2011). All-cause and bleeding-related health care costs in warfarin-treated patients with atrial fibrillation.. J Manag Care Pharm.

[17] Björholt I, Andersson S, Nilsson GH, Krakau I. (2007). The cost of monitoring warfarin in patients with chronic atrial fibrillation in primary care in Sweden.. BMC Fam Pract.

