# Supplementary material for: Recurrent Hospitalization Among Patients With Atrial Fibrillation Undergoing Intracoronary Stenting Treated With 2 Treatment Strategies of Rivaroxaban or a Dose-Adjusted Oral Vitamin K Antagonist Treatment Strategy
Source: Circulation. 2017 Jan 23;135(4):323–33. doi: 10.1161/CIRCULATIONAHA.116.025783 (PMC5266420; doi:10.1161/CIRCULATIONAHA.116.025783)
Supplement: Supplementary file 1 [file cir-135-323-s001.pdf]

SUPPLEMENTAL MATERIAL

Table S1. Line Listing of all adverse events requiring hospitalization and their categorization as either bleeding, cardiovascular or other

Adverse event leading to rehospitalization	Category	Frequency Count	Percent of Total Frequency
Abdominal Pain	Other	4	0.4
Abdominal Pain Lower	Other	1	0.1
Abdominal Wall Abscess	Other	2	0.2
Abscess Limb	Other	1	0.1
Acute Abdomen	Other	1	0.1
Acute Coronary Syndrome	Cardiovascular	4	0.4
Acute Kidney Injury	Other	5	0.5
Acute Myocardial Infarction	Cardiovascular	12	1.1
Acute Pulmonary Oedema	Cardiovascular	3	0.3
Acute Respiratory Failure	Cardiovascular	2	0.2
Adrenal Neoplasm	Other	1	0.1
Age-Related Macular Degeneration	Other	1	0.1
Ameloblastoma	Other	1	0.1
Anaemia	Bleeding	10	0.9
Anal Fistula	Other	1	0.1
Anal Haemorrhage	Bleeding	2	0.2
Anaphylactic Shock	Other	1	0.1
Angina Pectoris	Cardiovascular	40	3.7
Angina Unstable	Cardiovascular	53	4.9
Angioplasty	Cardiovascular	1	0.1
Anxiety	Other	1	0.1
Anxiety Disorder	Other	1	0.1
Aortic Aneurysm	Cardiovascular	1	0.1
Aortic Stenosis	Cardiovascular	3	0.3
Aortic Valve Replacement	Cardiovascular	1	0.1
Aphasia	Cardiovascular	1	0.1
Appendicitis	Other	2	0.2
Arterial Stenosis	Cardiovascular	2	0.2
Arteriosclerosis	Cardiovascular	1	0.1
Arteriovenous Fistula	Cardiovascular	1	0.1
Arthralgia	Other	3	0.3
Arthritis	Other	1	0.1

Adverse event leading to rehospitalization	Category	Frequency Count	Percent of Total Frequency
Arthritis Bacterial	Other	2	0.2
Atrial Fibrillation	Cardiovascular	70	6.5
Atrial Flutter	Cardiovascular	9	0.8
Atrial Tachycardia	Cardiovascular	4	0.4
Atrial Thrombosis	Cardiovascular	3	0.3
Atrioventricular Block	Cardiovascular	3	0.3
Atrioventricular Block Complete	Cardiovascular	1	0.1
Back Pain	Other	2	0.2
Bacterial Sepsis	Other	1	0.1
Barrett's Oesophagus	Other	1	0.1
Basal Cell Carcinoma	Other	1	0.1
Bile Duct Stone	Other	1	0.1
Biopsy Prostate	Other	1	0.1
Bladder Cancer	Other	1	0.1
Bladder Neoplasm	Other	1	0.1
Bladder Tamponade	Bleeding	1	0.1
Bowen's Disease	Other	2	0.2
Bradyarrhythmia	Cardiovascular	1	0.1
Bradycardia	Cardiovascular	5	0.5
Brain Neoplasm Malignant	Other	1	0.1
Bronchial Carcinoma	Other	1	0.1
Bronchitis	Other	5	0.5
Bronchitis Bacterial	Other	1	0.1
Bronchopneumopathy	Other	1	0.1
Bursitis	Other	1	0.1
Calculus Ureteric	Other	1	0.1
Calculus Urethral	Other	1	0.1
Calculus Urinary	Other	1	0.1
Cardiac Ablation	Cardiovascular	2	0.2
Cardiac Arrest	Cardiovascular	1	0.1
Cardiac Disorder	Cardiovascular	1	0.1
Cardiac Failure	Cardiovascular	74	6.9
Cardiac Failure Acute	Cardiovascular	8	0.7
Cardiac Failure Chronic	Cardiovascular	8	0.7
Cardiac Failure Congestive	Cardiovascular	25	2.3
Cardiac Pacemaker Insertion	Cardiovascular	1	0.1

Adverse event leading to rehospitalization	Category	Frequency Count	Percent of Total Frequency
Cardiac Pseudoaneurysm	Cardiovascular	1	0.1
Cardiac Resynchronisation Therapy	Cardiovascular	1	0.1
Cardiac Stress Test	Cardiovascular	1	0.1
Cardiogenic Shock	Cardiovascular	2	0.2
Cardiomyopathy	Cardiovascular	2	0.2
Cardiovascular Disorder	Cardiovascular	1	0.1
Cardioversion	Cardiovascular	2	0.2
Carotid Artery Stenosis	Cardiovascular	1	0.1
Cataract	Other	4	0.4
Catheter Site Swelling	Cardiovascular	1	0.1
Cellulitis	Other	4	0.4
Cerebral Haemorrhage	Bleeding	8	0.7
Cerebral Infarction	Cardiovascular	1	0.1
Cerebral Ischaemia	Cardiovascular	1	0.1
Cerebrovascular Accident	Cardiovascular	3	0.3
Cervical Polyp	Other	1	0.1
Change Of Bowel Habit	Other	1	0.1
Chest Discomfort	Cardiovascular	1	0.1
Chest Pain	Cardiovascular	22	2.0
Cholangiocarcinoma	Other	1	0.1
Cholangitis	Other	1	0.1
Cholecystectomy	Other	2	0.2
Cholecystitis	Other	3	0.3
Cholecystitis Acute	Other	3	0.3
Cholecystitis Chronic	Other	1	0.1
Cholelithiasis	Other	4	0.4
Chronic Obstructive Pulmonary Disease	Other	8	0.7
Circulatory Collapse	Cardiovascular	1	0.1
Clostridium Difficile Colitis	Other	1	0.1
Colon Cancer	Other	1	0.1
Colorectal Adenocarcinoma	Other	1	0.1
Compression Fracture	Other	1	0.1
Concussion	Bleeding	1	0.1
Confusional State	Cardiovascular	1	0.1
Constipation	Other	3	0.3
Contusion	Bleeding	1	0.1

Adverse event leading to rehospitalization	Category	Frequency Count	Percent of Total Frequency
Coronary Arterial Stent Insertion	Cardiovascular	1	0.1
Coronary Artery Bypass	Cardiovascular	1	0.1
Coronary Artery Disease	Cardiovascular	11	1.0
Coronary Artery Stenosis	Cardiovascular	2	0.2
Coronary Artery Thrombosis	Cardiovascular	1	0.1
Craniocerebral Injury	Cardiovascular	1	0.1
Crohn's Disease	Other	1	0.1
Cystitis	Other	1	0.1
Deafness Neurosensory	Other	1	0.1
Deep Vein Thrombosis	Cardiovascular	1	0.1
Dehydration	Other	2	0.2
Delirium	Cardiovascular	1	0.1
Depression	Other	1	0.1
Device Failure	Cardiovascular	1	0.1
Diabetes Mellitus	Other	3	0.3
Diabetic Foot	Other	1	0.1
Diabetic Metabolic Decompensation	Other	1	0.1
Diarrhoea	Other	2	0.2
Diffuse Large B-Cell Lymphoma	Other	1	0.1
Diverticulitis	Other	1	0.1
Dizziness	Cardiovascular	2	0.2
Dizziness Postural	Cardiovascular	1	0.1
Drug Intolerance	Other	1	0.1
Drug Withdrawal Syndrome	Other	1	0.1
Dysphagia	Other	1	0.1
Dyspnoea	Cardiovascular	27	2.5
Dyspnoea At Rest	Cardiovascular	1	0.1
Dyspnoea Exertional	Cardiovascular	4	0.4
Ejection Fraction Decreased	Cardiovascular	1	0.1
Electrocardiogram Ambulatory Abnormal	Cardiovascular	1	0.1
Encephalitis Viral	Other	1	0.1
Encephalopathy	Other	1	0.1
Enteritis	Other	1	0.1
Enterocolitis	Other	1	0.1
Epididymitis	Other	1	0.1
Epilepsy	Other	1	0.1

Adverse event leading to rehospitalization	Category	Frequency Count	Percent of Total Frequency
Epistaxis	Bleeding	16	1.5
Erysipelas	Other	1	0.1
Exostosis	Other	1	0.1
Extremity Necrosis	Cardiovascular	1	0.1
Eye Haemorrhage	Bleeding	1	0.1
Faecaloma	Other	1	0.1
Fall	Other	4	0.4
Fatigue	Cardiovascular	1	0.1
Femoral Artery Occlusion	Cardiovascular	2	0.2
Femoral Neck Fracture	Other	2	0.2
Gait Disturbance	Cardiovascular	1	0.1
Gangrene	Cardiovascular	3	0.3
Gastric Haemorrhage	Bleeding	1	0.1
Gastric Mucosa Erythema	Other	1	0.1
Gastric Ulcer	Bleeding	6	0.6
Gastric Ulcer Haemorrhage	Bleeding	1	0.1
Gastritis	Other	4	0.4
Gastritis Erosive	Bleeding	4	0.4
Gastritis Haemorrhagic	Bleeding	3	0.3
Gastroduodenitis Haemorrhagic	Bleeding	1	0.1
Gastroenteritis	Other	4	0.4
Gastrointestinal Haemorrhage	Bleeding	21	2.0
Gastrointestinal Infection	Other	1	0.1
Gastrointestinal Stromal Tumour	Other	1	0.1
Gastrointestinal Ulcer	Bleeding	1	0.1
Gastrooesophageal Reflux Disease	Other	2	0.2
Gouty Arthritis	Other	2	0.2
Haemarthrosis	Bleeding	1	0.1
Haematochezia	Bleeding	2	0.2
Haematoma	Bleeding	3	0.3
Haematoma Infection	Bleeding	1	0.1
Haematuria	Bleeding	11	1.0
Haemoglobin Decreased	Bleeding	1	0.1
Haemoptysis	Bleeding	8	0.7
Haemorrhage	Bleeding	2	0.2
Haemorrhage Urinary Tract	Bleeding	1	0.1

Adverse event leading to rehospitalization	Category	Frequency Count	Percent of Total Frequency
Haemorrhagic Anaemia	Bleeding	1	0.1
Haemorrhagic Stroke	Bleeding	2	0.2
Haemorrhoidal Haemorrhage	Bleeding	1	0.1
Haemorrhoids	Other	1	0.1
Haemothorax	Bleeding	1	0.1
Hallucination	Other	1	0.1
Hemiparesis	Cardiovascular	1	0.1
Hepatic Cyst	Other	1	0.1
Hepatitis Acute	Other	1	0.1
Hepatocellular Carcinoma	Other	1	0.1
Herpes Zoster	Other	1	0.1
Hiatus Hernia	Other	1	0.1
Hip Fracture	Other	1	0.1
Hospitalisation	Other	1	0.1
Humerus Fracture	Other	2	0.2
Hydronephrosis	Other	2	0.2
Hypergammaglobulinaemia	Other	1	0.1
Hyperglycaemia	Other	2	0.2
Hypertension	Cardiovascular	7	0.7
Hypertensive Crisis	Cardiovascular	7	0.7
Hyperthyroidism	Other	1	0.1
Hypoaesthesia	Other	1	0.1
Hypochromic Anaemia	Bleeding	1	0.1
Hypokalaemia	Other	1	0.1
Hyponatraemia	Cardiovascular	1	0.1
Hypotension	Cardiovascular	1	0.1
Ileostomy Closure	Other	1	0.1
Impaired Gastric Emptying	Other	1	0.1
Implantable Defibrillator Insertion	Cardiovascular	4	0.4
Incisional Hernia	Other	1	0.1
Infected Skin Ulcer	Other	1	0.1
Infective Exacerbation Of Chronic Obstructive Airways Disease	Other	3	0.3
Influenza	Other	2	0.2
Inguinal Hernia	Other	4	0.4
Intercostal Neuralgia	Other	1	0.1

Adverse event leading to rehospitalization	Category	Frequency Count	Percent of Total Frequency
Intermittent Claudication	Cardiovascular	3	0.3
International Normalised Ratio Increased	Cardiovascular	3	0.3
Interstitial Lung Disease	Other	1	0.1
Intervertebral Disc Protrusion	Other	1	0.1
Intestinal Ischaemia	Cardiovascular	2	0.2
Intestinal Obstruction	Other	1	0.1
Intra-Abdominal Haematoma	Bleeding	1	0.1
Intracranial Haematoma	Bleeding	1	0.1
Ischaemic Cardiomyopathy	Cardiovascular	1	0.1
Ischaemic Stroke	Cardiovascular	9	0.8
Jaundice	Other	1	0.1
Joint Effusion	Other	1	0.1
Knee Arthroplasty	Other	3	0.3
Lacunar Infarction	Cardiovascular	2	0.2
Left Ventricular Dysfunction	Cardiovascular	2	0.2
Left Ventricular Failure	Cardiovascular	2	0.2
Liver Abscess	Other	1	0.1
Localised Infection	Other	1	0.1
Lower Gastrointestinal Haemorrhage	Bleeding	2	0.2
Lower Limb Fracture	Other	1	0.1
Lower Respiratory Tract Infection	Other	1	0.1
Lumbar Spinal Stenosis	Other	1	0.1
Lung Neoplasm Malignant	Other	2	0.2
Lymphocele	Other	1	0.1
Malaise	Cardiovascular	1	0.1
Malignant Hypertension	Cardiovascular	2	0.2
Mallory-Weiss Syndrome	Bleeding	1	0.1
Medical Device Complication	Cardiovascular	1	0.1
Melaena	Bleeding	6	0.6
Meningioma	Other	1	0.1
Metabolic Disorder	Other	1	0.1
Microcytic Anaemia	Bleeding	2	0.2
Mitral Valve Repair	Cardiovascular	1	0.1
Mouth Haemorrhage	Bleeding	1	0.1
Multi-Organ Failure	Cardiovascular	1	0.1
Muscle Rupture	Other	1	0.1

Adverse event leading to rehospitalization	Category	Frequency Count	Percent of Total Frequency
Musculoskeletal Disorder	Other	1	0.1
Myocardial Infarction	Cardiovascular	2	0.2
Myocardial Ischaemia	Cardiovascular	3	0.3
Nasopharyngitis	Other	1	0.1
Nausea	Other	1	0.1
Neck Pain	Other	1	0.1
Neoplasm	Other	1	0.1
Neuralgia	Other	1	0.1
Neurodermatitis	Other	1	0.1
Non-Cardiac Chest Pain	Cardiovascular	4	0.4
Oedema Peripheral	Cardiovascular	2	0.2
Osteoarthritis	Other	5	0.5
Osteochondrosis	Other	1	0.1
Overdose	Other	5	0.5
Pain In Extremity	Other	2	0.2
Palpitations	Cardiovascular	3	0.3
Pancreatitis	Other	3	0.3
Pancreatitis Chronic	Other	1	0.1
Parkinson's Disease	Other	1	0.1
Percutaneous Coronary Intervention	Cardiovascular	1	0.1
Periarthritis	Other	1	0.1
Pericardial Effusion	Cardiovascular	1	0.1
Pericardial Haemorrhage	Bleeding	1	0.1
Pericarditis	Cardiovascular	1	0.1
Periodontitis	Other	1	0.1
Peripheral Arterial Occlusive Disease	Cardiovascular	6	0.6
Peripheral Artery Thrombosis	Cardiovascular	3	0.3
Peripheral Embolism	Cardiovascular	1	0.1
Peripheral Ischaemia	Cardiovascular	4	0.4
Peripheral Vascular Disorder	Cardiovascular	1	0.1
Pituitary Tumour Benign	Other	1	0.1
Pleural Effusion	Cardiovascular	1	0.1
Pneumonia	Other	37	3.4
Pneumothorax Traumatic	Other	1	0.1
Post Procedural Haematoma	Bleeding	1	0.1
Post Procedural Haemorrhage	Bleeding	2	0.2

Adverse event leading to rehospitalization	Category	Frequency Count	Percent of Total Frequency
Presyncope	Cardiovascular	1	0.1
Prostate Cancer	Other	2	0.2
Pubis Fracture	Other	1	0.1
Pulmonary Congestion	Cardiovascular	1	0.1
Pulmonary Hypertension	Cardiovascular	4	0.4
Pulmonary Mass	Other	1	0.1
Pulmonary Oedema	Cardiovascular	5	0.5
Pyelonephritis	Other	1	0.1
Pyrexia	Other	1	0.1
Radius Fracture	Other	1	0.1
Rectal Cancer	Other	2	0.2
Rectal Haemorrhage	Bleeding	8	0.7
Rectal Neoplasm	Other	1	0.1
Renal Artery Stenosis	Cardiovascular	1	0.1
Renal Cell Carcinoma	Other	1	0.1
Renal Failure	Other	2	0.2
Renal Impairment	Other	1	0.1
Renal Neoplasm	Other	1	0.1
Renal Sympathetic Nerve Ablation	Other	1	0.1
Respiratory Distress	Cardiovascular	1	0.1
Respiratory Failure	Cardiovascular	1	0.1
Respiratory Tract Infection	Other	2	0.2
Rhabdomyolysis	Other	1	0.1
Rheumatic Disorder	Other	1	0.1
Rib Fracture	Other	2	0.2
Road Traffic Accident	Other	1	0.1
Sciatica	Other	1	0.1
Scrotal Abscess	Other	1	0.1
Sepsis	Other	3	0.3
Septic Shock	Other	2	0.2
Sinus Bradycardia	Cardiovascular	1	0.1
Sinus Node Dysfunction	Cardiovascular	8	0.7
Skin Necrosis	Cardiovascular	2	0.2
Skin Ulcer	Other	1	0.1
Sleep Apnoea Syndrome	Other	3	0.3
Small Cell Lung Cancer Metastatic	Other	1	0.1

Adverse event leading to rehospitalization	Category	Frequency Count	Percent of Total Frequency
Small Intestinal Obstruction	Other	1	0.1
Spinal Column Stenosis	Other	1	0.1
Spinal Disorder	Other	1	0.1
Spinal Osteoarthritis	Other	1	0.1
Spinal Pain	Other	1	0.1
Squamous Cell Carcinoma	Other	1	0.1
Stomatitis	Other	1	0.1
Subdural Haematoma	Bleeding	2	0.2
Subdural Haemorrhage	Bleeding	2	0.2
Supraventricular Tachycardia	Cardiovascular	3	0.3
Syncope	Cardiovascular	15	1.4
Tachyarrhythmia	Cardiovascular	1	0.1
Tachycardia Induced Cardiomyopathy	Cardiovascular	1	0.1
Tension Headache	Other	1	0.1
Tongue Haemorrhage	Bleeding	1	0.1
Tooth Extraction	Other	1	0.1
Tracheitis	Other	1	0.1
Transient Ischaemic Attack	Cardiovascular	5	0.5
Traumatic Fracture	Other	2	0.2
Traumatic Haematoma	Bleeding	1	0.1
Tuberculosis	Other	1	0.1
Ulcer Haemorrhage	Bleeding	1	0.1
Upper Gastrointestinal Haemorrhage	Bleeding	4	0.4
Upper Limb Fracture	Other	1	0.1
Upper Respiratory Tract Infection	Other	1	0.1
Urethral Haemorrhage	Bleeding	1	0.1
Urethral Stenosis	Other	1	0.1
Urinary Retention	Other	2	0.2
Urinary Tract Infection	Other	6	0.6
Urosepsis	Other	1	0.1
Urticaria	Other	1	0.1
Vascular Pseudoaneurysm	Cardiovascular	2	0.2
Vascular Stent Occlusion	Cardiovascular	1	0.1
Vascular Stent Restenosis	Cardiovascular	2	0.2
Vascular Stent Thrombosis	Cardiovascular	3	0.3
Venous Thrombosis	Cardiovascular	1	0.1

Adverse event leading to rehospitalization	Category	Frequency Count	Percent of Total Frequency
Ventricular Extrasystoles	Cardiovascular	1	0.1
Ventricular Fibrillation	Cardiovascular	4	0.4
Ventricular Tachycardia	Cardiovascular	3	0.3
Vertigo	Other	1	0.1
Vessel Puncture Site Haematoma	Bleeding	1	0.1
Viral Upper Respiratory Tract Infection	Other	1	0.1
Vitreous Haemorrhage	Bleeding	1	0.1
Wound Dehiscence	Other	1	0.1

Table S2: Baseline Characteristics (Cont.)

Characteristic	**Group 1** Rivaroxaban + $P2Y_{12}$ (N=709)	**Group 2** Rivaroxaban + DAPT (N=709)	**Group 3** VKA + DAPT (N=706)
BMI, median (IQR) †	28.6 (25.7 – 32.4)	28.4 (25.6 – 32.1)	29.0 (25.8 – 32.8)
Urgency of Revascularization – no. (%)			
Elective	428 (60.4)	430 (60.6)	449 (63.6)
Urgent	281 (39.6)	279 (39.4)	257 (36.4)
$CHADS_2$ risk of stroke – no. (%)			
0	99 (14.0)	90 (12.7)	83 (11.8)
1	220 (31.0)	232 (32.7)	227 (32.2)
2	246 (34.7)	256 (36.1)	273 (38.7)
3	128 (18.1)	118 (16.6)	107 (15.2)
4	16 (2.3)	13 (1.8)	16 (2.3)
5	0 (0.0)	0 (0.0)	0 (0.0)
6	0 (0.0)	0 (0.0)	0 (0.0)
HAS Bled Score – no. (%)			
0	2 (0.3)	2 (0.3)	0 (0.0)
1	28 (4.0)	43 (6.1)	26 (3.7)
2	166 (23.4)	182 (25.7)	182 (25.8)
3	321 (45.3)	294 (41.5)	308 (43.6)
4	160 (22.6)	157 (22.1)	157 (22.2)
5	31 (4.4)	30 (4.2)	31 (4.4)
6	1 (0.1)	1 (0.1)	2 (0.3)
Comorbidities – no. (%)			
Congestive heart failure	180 (25.4)	187 (26.4)	175 (24.8)
Hypertension	520 (73.3)	519 (73.2)	532 (75.4)
Diabetes mellitus	204 (28.8)	199 (28.1)	221 (31.3)
Hypercholesterolemia	302 (42.6)	295 (41.6)	316 (44.8)
Previous myocardial infarction	140 (19.8)	180 (25.4)	157 (22.2)
Peripheral vascular disease	30 (4.2)	42 (5.9)	35 (5.0)
Gastrointestinal bleeding	7 (1.0)	9 (1.3)	5 (0.7)
Medications – no. (%)			
Aspirin[¥]	9 (1.3)	702 (99.7)	699 (99.6)
Beta-blocker	586 (82.7)	541 (76.3)	537 (76.1)
ACE inhibitor or ARB	571 (80.5)	532 (75.0)	537 (76.1)
Statin	596 (84.1)	557 (78.6)	552 (78.2)
Proton pump inhibitor			
Omeprazole or esomeprazole	74 (10.4)	78 (11.0)	79 (11.2)
Other	200 (28.2)	198 (27.9)	180 (25.5)

[¥] Aspirin use was calculated as administration of aspirin no more than 4 days after PCI procedure for index event.
There were significant differences across groups in the following categories; previous myocardial infarction (p=0.039 overall, p=0.011 Group 1 v Group 2), aspirin use (p<0.001 overall, p<0.001 Group 1 v Group 2, p<0.001 Group 1 v Group 3), beta-blocker use (p=0.003 overall, p=0.002 Group 1 v Group 3, p=0.003 Group 1 v Group 2), ACE inhibitor or ARB use (p=0.032 overall, p=0.042 Group 1 v Group 3, p=0.013 Group 1 v Group 2) and statin use (p=0.008 overall, p=0.005 Group 1 v Group 3, p=0.008 Group 1 v Group 2). All other p-values were not significant.
Note: Plus-minus values are mean ± SD. There were no significant differences among the three groups. ACE denotes angiotensin-converting enzyme, ARB angiotensin-receptor blocker, BMI denotes body mass index.
Note: Numbers based upon all randomized subjects.
Note: Pairwise comparisons were calculated using the chi-square test of independence for categorical variables, independent samples t-test for parametric continuous variables, and Wilcoxon rank sum test for non-parametric continuous variables.

Table S3: Rate of Events Leading to Recurrent Hospitalization by Severity

Endpoint	Group 1 (N= 696)	Group 2 (N=706)	Group 3 (N=697)	Group 1 vs. Group 3 Rivaroxaban + $P2Y_{12}$ vs. VKA + DAPT		Group 2 vs. Group 3 Rivaroxaban + DAPT vs. VKA + DAPT	
				**HR (95% CI)**	**p-value**	**HR (95% CI)**	**p-value**
**Overall**	**221 (34.1)**	**207 (31.2)**	**257 (41.5)**	**0.77 (0.65 – 0.92)**	**0.005**	**0.74 (0.61 – 0.88)**	**0.001**
Severe	70 (11.1)	74 (11.4)	83 (13.8)	0.78 (0.57 – 1.08)	0.13	0.84 (0.62 – 1.15)	0.28
Moderate	142 (22.7)	120 (18.7)	163 (27.3)	0.80 (0.64 – 1.00)	0.05	0.68 (0.54 – 0.86)	0.001
Mild	53 (8.6)	54 (8.5)	56 (9.8)	0.88 (0.61 – 1.28)	0.51	0.90 (0.62 – 1.30)	0.56
**Bleeding or Cardiovascular**	**159 (24.7)**	**158 (24.0)**	**219 (35.7)**	**0.64 (0.52 – 0.79)**	**<0.001**	**0.66 (0.54 – 0.81)**	**<0.001**
Severe	51 (8.2)	56 (8.6)	64 (10.6)	0.74 (0.51 – 1.07)	0.11	0.84 (0.59 – 1.20)	0.33
Moderate	99 (15.9)	84 (13.1)	135 (22.7)	0.67 (0.52 – 0.87)	0.002	0.57 (0.44 – 0.75)	<0.001
Mild	30 (4.8)	43 (6.8)	46 (8.0)	0.61 (0.38 – 0.96)	0.031	0.87 (0.57 – 1.32)	0.51
**Bleeding**	**41 (6.5)**	**34 (5.4)**	**63 (10.5)**	**0.61 (0.41 – 0.90)**	**0.012**	**0.51 (0.34 – 0.77)**	**0.001**
Severe	12 (1.9)	8 (1.3)	25 (4.2)	0.45 (0.23 – 0.90)	0.021	0.34 (0.16 – 0.72)	0.003
Moderate	23 (3.7)	20 (3.2)	33 (5.7)	0.65 (0.38 – 1.11)	0.12	0.56 (0.32 – 0.97)	0.037
Mild	6 (1.0)	7 (1.1)	6 (1.0)	0.95 (0.31 – 2.93)	0.92	1.09 (0.37 – 3.24)	0.88
**Cardiovascular**	**128 (20.3)**	**133 (20.3)**	**169 (28.4)**	**0.68 (0.54 – 0.85)**	**<0.001**	**0.73 (0.58 – 0.91)**	**0.005**
Severe	39 (6.3)	48 (7.4)	41 (6.9)	0.89 (0.57 – 1.38)	0.59	1.10 (0.73 – 1.67)	0.64
Moderate	80 (12.9)	66 (10.3)	107 (18.3)	0.68 (0.51 – 0.91)	0.009	0.57 (0.42 – 0.78)	<0.001
Mild	26 (4.2)	41 (6.5)	40 (7.0)	0.61 (0.37 – 0.99)	0.044	0.95 (0.62 – 1.48)	0.83
**Other**	**91 (14.8)**	**74 (11.7)**	**83 (14.3)**	**1.04 (0.77 – 1.40)**	**0.79**	**0.82 (0.60 – 1.13)**	**0.22**
Severe	21 (3.4)	23 (3.7)	23 (4.0)	0.86 (0.48 – 1.55)	0.62	0.92 (0.52 – 1.64)	0.78
Moderate	60 (9.9)	42 (6.8)	51 (8.8)	1.11 (0.77 – 1.62)	0.58	0.76 (0.51 – 1.15)	0.19
Mild	27 (4.5)	13 (2.1)	13 (2.3)	1.96 (1.01 – 3.80)	0.042	0.93 (0.43 – 2.00)	0.85

Note: Treatment-emergent period: period starting after the first study drug administration following randomization and ending 2 days after stop of study drug.
Note: Event rate of first rehospitalization up to 360 days of study duration is calculated by the Kaplan-Meier method.
Note: Hazard ratios as compared to the VKA group are based on the Cox proportional hazards model.

Note: Rehospitalization is defined as the hospital admission after the first index event.
Note: Log-rank P-values as compared to VKA group are based on the two-sided log-rank test.
Note: An assessment of severity grade will be made using the following general categorical descriptors: a) *Mild*: Awareness of symptoms that are easily tolerated, causing minimal discomfort and not interfering with everyday activities; b) *Moderate*: Sufficient discomfort is present to cause interference with normal activity; c) *Severe*: Extreme distress, causing significant impairment of functioning or incapacitation and preventing normal everyday activities.

Table S4: Power calculation assuming a risk reduction of ≥ 20 % at a two-sided significance level of 0.05

Current Substudy			Main Study		
**Endpoint**	**Event rate**	**Power**	**Endpoint**	**Event rate**	**Power**
**Overall**					
Death or rehospitalization	41.9%	90.0%	Adverse CV event	6.0%	16.8%
Death or bleeding / cardiovascular rehosp.	36.4%	82.8%			
**DAPT 1 month**					
Death or rehospitalization	51.2%	97.1%	Adverse CV event	5.1%	14.9%
Death or bleeding / cardiovascular rehosp.	42.6%	90.7%			
**DAPT 6 months**					
Death or rehospitalization	42.7%	90.8%	Adverse CV event	4.3%	13.3%
Death or bleeding / cardiovascular rehosp.	38.6%	85.9%			
**DAPT 12 months**					
Death or rehospitalization	38.2%	85.4%	Adverse CV event	7.4%	19.9%
Death or bleeding / cardiovascular rehosp.	32.8%	76.8%			

Note: Power was calculated based on the observed event rate in the VKA arm using the Pearson’s chi-square test.
Note: Both the treatment and control arms are standardized to 700 subjects.

Table S5: Baseline Characteristics by DAPT Stratum for Subjects (Group 2 or Group 3)

Characteristic	DAPT 1 Month (N = 224)	DAPT 6 Months (N = 494)	DAPT 12 Months (N = 697)	p-value
**Demographics**				
Age				
Mean ─ yr	71.7 ± 8.7	69.9 ± 8.7	69.4 ± 9.0	0.003
≥ 65 yr ─ no. (%)	176 (78.6)	368 (74.5)	498 (71.5)	0.095
≥ 75 yr ─ no. (%)	95 (42.4)	163 (33.0)	217 (31.1)	0.008
Female sex ─ no. (%)	48 (21.4)	127 (25.7)	187 (26.8)	0.272
Race*─ no. (%)				0.172
White	216 (96.4)	457 (92.5)	662 (95.0)	
Black or African-American	1 (0.5)	1 (0.2)	2 (0.3)	
Asian	5 (2.2)	31 (6.3)	25 (3.6)	
American Indian or Alaska Native	0 (0.0)	0 (0.0)	0 (0.0)	
Other or unknown	2 (0.9)	5 (1.0)	8 (1.2)	
BMI†				
Median	27.8	29.1	28.6	0.259
Interquartile range	25.4 – 32.5	25.8 – 32.6	25.8 – 32.4	
Active smokers – no. (%)	13 (5.8)	37 (7.5)	54 (7.8)	0.618
Creatinine clearance ─ ml/min‡				
Mean	75.9 ± 33.4	79.6 ± 29.8	79.8 ± 30.9	0.255
< 60 to ≥ 30 ml/min ─ no. (%)	68 (31.9)	138 (28.9)	165 (25.1)	0.104
<30 ml/min ─ no. (%)	2 (0.9)	1 (0.2)	6 (0.9)	0.276
P2Y12 inhibitor at baseline – no. (%)				0.056
Clopidogrel	221 (98.7)	467 (94.5)	656 (94.1)	
Prasugrel	1 (0.5)	4 (0.8)	11 (1.6)	
Ticagrelor	2 (0.9)	23 (4.7)	30 (4.3)	
**Index Event**				
Type of Index Event – no. (%)				0.089
NSTEMI	28 (12.8)	96 (19.6)	128 (18.6)	
STEMI	18 (8.3)	65 (13.3)	88 (12.8)	
Unstable Angina	53 (24.3)	107 (21.9)	152 (22.1)	
Stable Angina	119 (54.6)	221 (45.2)	319 (46.4)	
Type of Stent – no. (%)				<0.001
Drug-eluting stent	67 (29.9)	374 (75.9)	498 (72.0)	
Bare metal stent	156 (69.6)	114 (23.1)	174 (25.1)	
Drug-eluting and bare metal stents	1 (0.5)	5 (1.0)	20 (2.9)	
Urgency of Revascularization – no. (%)				<0.001
Elective	157 (70.1)	324 (65.6)	398 (57.1)	
Urgent	67 (29.9)	170 (34.4)	299 (42.9)	
Type of Atrial Fibrillation ─ no. (%)				0.037

Characteristic	DAPT 1 Month (N = 224)	DAPT 6 Months (N = 494)	DAPT 12 Months (N = 697)	p-value
Persistent	52 (23.2)	121 (24.5)	122 (17.5)	
Permanent	78 (34.8)	154 (31.2)	249 (35.8)	
Paroxysmal	94 (42.0)	219 (44.3)	325 (46.7)	
**Bleed Risk Scores**				
$CHADS_2$ risk of stroke – no. (%)				0.270
0	28 (12.5)	53 (10.7)	92 (13.2)	
1	74 (33.0)	147 (29.8)	238 (34.2)	
2	82 (36.6)	204 (41.3)	243 (34.9)	
3	32 (14.3)	81 (16.4)	112 (16.1)	
4	8 (27.6)	9 (1.8)	12 (1.7)	
5	0 (0.0)	0 (0.0)	0 (0.0)	
6	0 (0.0)	0 (0.0)	0 (0.0)	
CHA_2DS_2-VASc risk of stroke – no. (%)				0.457
0	3 (1.3)	4 (0.8)	10 (1.4)	
1	16 (7.1)	32 (6.5)	61 (8.8)	
2	34 (15.2)	55 (11.1)	100 (14.4)	
3	45 (20.1)	92 (18.6)	133 (19.1)	
4	48 (21.4)	121 (24.5)	158 (22.7)	
5	43 (19.2)	103 (20.9)	142 (20.4)	
6	32 (14.3)	72 (14.6)	72 (10.3)	
7	3 (1.3)	15 (3.0)	21 (3.0)	
HAS Bled Score – no. (%)				0.031
0	0 (0.0)	1 (0.2)	1 (0.1)	
1	8 (3.6)	15 (3.0)	46 (6.6)	
2	46 (20.5)	128 (25.9)	190 (27.3)	
3	103 (46.0)	200 (40.5)	299 (42.9)	
4	54 (24.1)	122 (24.7)	138 (19.8)	
5	13 (5.8)	27 (5.5)	21 (3.0)	
6	0 (0.0)	1 (0.2)	2 (0.3)	
**Comorbidities**				
Congestive heart failure	46 (20.5)	135 (27.3)	181 (26.0)	0.147
Hypertension	165 (73.7)	508 (72.9)	378 (76.5)	0.359
Diabetes mellitus	60 (26.8)	158 (32.0)	202 (29.0)	0.314
Hypercholesterolemia	97 (43.3)	225 (45.6)	289 (41.5)	0.374
Previous myocardial infarction	47 (21.0)	111 (22.5)	179 (25.7)	0.244
Peripheral vascular disease	11 (4.9)	27 (5.5)	39 (5.6)	0.925
Gastrointestinal bleeding	1 (0.5)	4 (0.8)	9 (1.3)	0.620
**Medications**				
Aspirin[¥]	222 (99.1)	493 (99.8)	695 (99.7)	0.309
Beta-blocker	175 (78.1)	375 (75.9)	528 (75.8)	0.757
ACE inhibitor or ARB	168 (75.0)	385 (77.9)	516 (74.0)	0.297

Characteristic	DAPT 1 Month (N = 224)	DAPT 6 Months (N = 494)	DAPT 12 Months (N = 697)	p-value
Statin	164 (73.2)	374 (75.7)	571 (81.9)	0.005
Proton pump inhibitor				0.406
Omeprazole or esomeprazole	27 (12.1)	53 (10.7)	77 (11.1)	
Other	59 (26.3)	118 (23.9)	200 (28.7)	

*Race was self-reported.
† Body mass index (BMI) is the weight (kg) divided by the square of the height (m).
‡ Creatinine clearance calculated using the Cockcroft-Gault equation.
¥ Aspirin use was calculated as administration of aspirin no more than 4 days after PCI procedure for index event.
Note: Plus-minus values are mean ± SD. ACE denotes angiotensin-converting enzyme, ACS acute coronary syndrome, ARB angiotensin-receptor blocker, BMI denotes body mass index, PCI percutaneous coronary intervention, NSTEMI non-ST-segment elevation myocardial infarction, STEMI ST-segment elevation myocardial infarction.
Note: Numbers based upon all randomized subjects.
Note: Comparisons were calculated using the chi-square test of independence for categorical variables, ANOVA for parametric continuous variables, and Wilcoxon rank sum test for non-parametric continuous variables.

Table S6: Baseline Characteristics by DAPT Duration (1 month) and Treatment

Characteristic	Group 2<br>Rivaroxaban + DAPT<br>(N = 109)	Group 3<br>VKA + DAPT<br>(N = 115)	p-value
**Demographics**			
Age			
Mean ─ yr	70.8 ± 9.6	72.6 ± 7.8	0.126
≥ 65 yr ─ no. (%)	80 (73.4)	96 (83.5)	0.066
≥ 75 yr ─ no. (%)	44 (40.4)	51 (44.4)	0.547
Female sex ─ no. (%)	24 (22.0)	24 (20.9)	0.834
Race*─ no. (%)			0.791
White	105 (96.3)	111 (96.5)	
Black or African-American	0 (0.0)	1 (0.9)	
Asian	2 (1.8)	3 (2.6)	
American Indian or Alaska Native	0 (0.0)	0 (0.0)	
Other or unknown	2 (1.8)	0 (0.0)	
BMI†			
Median	27.8	27.7	0.654
Interquartile range	25.5 – 33.2	25.4 – 32.0	
Active smokers – no. (%)	5 (4.6)	8 (7.0)	0.448
Creatinine clearance ─ ml/min‡			
Mean	78.3 ± 37.1	73.5 ± 29.3	0.302
< 60 to ≥ 30 ml/min ─ no. (%)	28 (26.7)	40 (37.0)	0.105
<30 ml/min ─ no. (%)	2 (1.9)	0 (0.0)	0.242
P2Y12 inhibitor at baseline – no. (%)			0.236
Clopidogrel	107 (98.2)	114 (99.1)	
Prasugrel	0 (0.0)	1 (0.9)	
Ticagrelor	2 (1.8)	0 (0.0)	
**Index Event**			
Type of Index Event – no. (%)			0.385
NSTEMI	16 (14.8)	12 (10.9)	
STEMI	9 (8.3)	9 (8.2)	
Unstable Angina	21 (19.4)	32 (29.1)	
Stable Angina	62 (57.4)	57 (51.8)	
Type of Stent – no. (%)			0.611
Drug-eluting stent	34 (31.2)	33 (28.7)	
Bare metal stent	74 (67.9)	82 (71.3)	
Drug-eluting and bare metal stents	1 (0.9)	0 (0.0)	
Urgency of Revascularization – no. (%)			0.908
Elective	76 (69.7)	81 (70.4)	
Urgent	33 (30.3)	34 (29.6)	
Type of Atrial Fibrillation ─ no. (%)			0.176

Characteristic	**Group 2** Rivaroxaban + DAPT (N = 109)	**Group 3** VKA + DAPT (N = 115)	p-value
Persistent	20 (18.4)	32 (27.8)	
Permanent	43 (39.5)	35 (30.4)	
Paroxysmal	46 (42.2)	48 (41.7)	
**Bleed Risk Scores**			
$CHADS_2$ risk of stroke – no. (%)			0.184
0	16 (14.7)	12 (10.4)	
1	39 (35.8)	35 (30.4)	
2	32 (29.4)	50 (43.5)	
3	19 (17.4)	13 (11.3)	
4	3 (2.8)	5 (4.4)	
5	0 (0.0)	0 (0.0)	
6	0 (0.0)	0 (0.0)	
CHA_2DS_2-VASc risk of stroke – no. (%)			0.180
0	2 (1.8)	1 (0.9)	
1	12 (11.0)	4 (3.5)	
2	13 (11.9)	21 (18.3)	
3	19 (17.4)	26 (22.6)	
4	20 (18.4)	28 (24.4)	
5	23 (21.1)	20 (17.4)	
6	19 (17.4)	13 (11.3)	
7	1 (0.9)	2 (1.7)	
HAS Bled Score – no. (%)			0.671
0	0 (0.0)	0 (0.0)	
1	5 (4.6)	3 (2.6)	
2	23 (21.1)	23 (20.0)	
3	45 (41.3)	58 (50.4)	
4	29 (26.6)	25 (21.7)	
5	7 (6.4)	6 (5.2)	
6	0 (0.0)	0 (0.0)	
**Comorbidities**			
Congestive heart failure	21 (19.3)	25 (21.7)	0.647
Hypertension	77 (70.6)	88 (76.5)	0.318
Diabetes mellitus	30 (27.5)	30 (26.1)	0.808
Hypercholesterolemia	38 (34.9)	59 (51.3)	0.013
Previous myocardial infarction	19 (17.4)	28 (24.4)	0.204
Peripheral vascular disease	7 (6.4)	4 (3.5)	0.308
Gastrointestinal bleeding	0 (0.0)	1 (0.9)	>0.999
**Medications**			
Aspirin[¥]	108 (99.1)	114 (99.1)	>0.999
Beta-blocker	87 (79.8)	88 (76.5)	0.551
ACE inhibitor or ARB	83 (76.2)	85 (73.9)	0.700

Characteristic	Group 2<br>Rivaroxaban + DAPT<br>(N = 109)	Group 3<br>VKA + DAPT<br>(N = 115)	p-value
Statin	79 (72.5)	85 (73.9)	0.808
Proton pump inhibitor			0.107
Omeprazole or esomeprazole	9 (8.3)	18 (15.7)	
Other	34 (31.2)	25 (21.7)	

*Race was self-reported.
† Body mass index (BMI) is the weight (kg) divided by the square of the height (m).
‡ Creatinine clearance calculated using the Cockcroft-Gault equation.
¥ Aspirin use was calculated as administration of aspirin no more than 4 days after PCI procedure for index event.
Note: Plus-minus values are mean ± SD. ACE denotes angiotensin-converting enzyme, ACS acute coronary syndrome, ARB angiotensin-receptor blocker, BMI denotes body mass index, PCI percutaneous coronary intervention, NSTEMI non-ST-segment elevation myocardial infarction, STEMI ST-segment elevation myocardial infarction.
Note: Numbers based upon all randomized subjects and available data.
Note: Pairwise comparisons were calculated using the chi-square test of independence for categorical variables, independent samples t-test for parametric continuous variables, and Wilcoxon rank sum test for non-parametric continuous variables.

Table S7: Baseline Characteristics by DAPT Duration (6 months) and Treatment

Characteristic	Group 2<br>Rivaroxaban + DAPT<br>(N = 248)	Group 3<br>VKA + DAPT<br>(N = 246)	p-value
**Demographics**			
Age			
Mean ─ yr	70.2 ± 9.1	69.6 ± 8.3	0.403
≥ 65 yr ─ no. (%)	183 (73.8)	185 (75.2)	0.719
≥ 75 yr ─ no. (%)	90 (36.3)	73 (29.7)	0.118
Female sex ─ no. (%)	65 (26.2)	62 (25.2)	0.798
Race*─ no. (%)			0.039
White	235 (94.8)	222 (90.2)	
Black or African-American	1 (0.4)	0 (0.0)	
Asian	11 (4.4)	20 (8.1)	
American Indian or Alaska Native	0 (0.0)	0 (0.0)	
Other or unknown	1 (0.4)	4 (1.6)	
BMI†			
Median	28.7	29.4	0.297
Interquartile range	25.7 – 32.3	25.8 – 32.8	
Active smokers – no. (%)	21 (8.5)	16 (6.5)	0.407
Creatinine clearance ─ ml/min‡			
Mean	77.5 ± 30.0	81.8 ± 29.5	0.114
< 60 to ≥ 30 ml/min ─ no. (%)	78 (32.2)	60 (25.5)	0.108
<30 ml/min ─ no. (%)	1 (0.4)	0 (0.0)	>0.999
$P2Y_{12}$ inhibitor at baseline – no. (%)			0.370
Clopidogrel	231 (93.2)	236 (95.9)	
Prasugrel	3 (1.2)	1 (0.4)	
Ticagrelor	14 (5.7)	9 (3.7)	
**Index Event**			
Type of Index Event – no. (%)			0.451
NSTEMI	51 (20.7)	45 (18.5)	
STEMI	38 (15.5)	27 (11.1)	
Unstable Angina	52 (21.1)	55 (22.6)	
Stable Angina	105 (42.7)	116 (47.7)	
Type of Stent – no. (%)			0.509
Drug-eluting stent	187 (75.7)	187 (76.0)	
Bare metal stent	56 (22.7)	58 (23.6)	
Drug-eluting and bare metal stents	4 (1.6)	1 (0.4)	
Urgency of Revascularization – no. (%)			0.101
Elective	154 (62.1)	170 (69.1)	
Urgent	94 (37.9)	76 (30.9)	

**Characteristic**	**Group 2** Rivaroxaban + DAPT (N = 248)	**Group 3** VKA + DAPT (N = 246)	**p-value**
Type of Atrial Fibrillation ─ no. (%)			0.932
Persistent	60 (24.2)	61 (24.8)	
Permanent	76 (30.7)	78 (31.7)	
Paroxysmal	112 (45.2)	107 (43.5)	
**Bleed Risk Scores**			
$CHADS_2$ risk of stroke – no. (%)			0.676
0	26 (10.5)	27 (11.0)	
1	74 (29.8)	73 (29.7)	
2	108 (43.6)	96 (39.0)	
3	35 (14.1)	46 (18.7)	
4	5 (2.0)	4 (1.6)	
5	0	0	
6	0	0	
CHA_2DS_2-VASc risk of stroke – no. (%)			0.381
0	2 (0.8)	2 (0.8)	
1	20 (8.1)	12 (4.9)	
2	24 (9.7)	31 (12.6)	
3	51 (20.6)	41 (16.7)	
4	55 (22.2)	66 (26.8)	
5	56 (22.6)	47 (19.1)	
6	35 (14.1)	37 (15.0)	
7	5 (2.0)	10 (4.1)	
HAS Bled Score – no. (%)			0.673
0	1 (0.4)	0 (0.0)	
1	10 (4.0)	5 (2.0)	
2	64 (25.8)	64 (26.0)	
3	100 (40.3)	100 (40.7)	
4	60 (24.2)	62 (25.2)	
5	12 (4.8)	15 (6.1)	
6	1 (0.4)	0 (0.0)	
**Comorbidities**			
Congestive heart failure	67 (27.0)	68 (27.6)	0.876
Hypertension	188 (75.8)	190 (77.2)	0.708
Diabetes mellitus	70 (28.2)	88 (35.8)	0.072
Hypercholesterolemia	109 (44.0)	116 (47.2)	0.475
Previous myocardial infarction	62 (25.0)	49 (19.9)	0.176
Peripheral vascular disease	12 (4.8)	15 (6.1)	0.538
Gastrointestinal bleeding	2 (0.8)	2 (0.8)	>0.999
**Medications**			
Aspirin[¥]	248 (100.0)	245 (99.6)	0.498
Beta-blocker	193 (77.8)	182 (74.0)	0.319

Characteristic	**Group 2**<br>Rivaroxaban + DAPT<br>(N = 248)	**Group 3**<br>VKA + DAPT<br>(N = 246)	p-value
ACE inhibitor or ARB	199 (80.2)	186 (75.6)	0.215
Statin	194 (78.2)	180 (73.2)	0.190
Proton pump inhibitor			0.108
Omeprazole or esomeprazole	30 (12.1)	23 (9.4)	
Other	67 (27.0)	51 (20.7)	

*Race was self-reported.
† Body mass index (BMI) is the weight (kg) divided by the square of the height (m).
‡ Creatinine clearance calculated using the Cockcroft-Gault equation.
¥ Aspirin use was calculated as administration of aspirin no more than 4 days after PCI procedure for index event.
Note: Plus-minus values are mean ± SD. ACE denotes angiotensin-converting enzyme, ACS acute coronary syndrome, ARB angiotensin-receptor blocker, BMI denotes body mass index, PCI percutaneous coronary intervention, NSTEMI non-ST-segment elevation myocardial infarction, STEMI ST-segment elevation myocardial infarction.
Note: Numbers based upon all randomized subjects and available data.
Note: Pairwise comparisons were calculated using the chi-square test of independence for categorical variables, independent samples t-test for parametric continuous variables, and Wilcoxon rank sum test for non-parametric continuous variables.

Table S8: Baseline Characteristics by DAPT Duration (12 months) and Treatment

Characteristic	Group 2<br>Rivaroxaban + DAPT<br>(N = 352)	Group 3<br>VKA + DAPT<br>(N = 345)	p-value
**Demographics**			
Age			
Mean ─ yr	69.5 ± 9.0	69.3 ± 9.1	0.721
≥ 65 yr ─ no. (%)	253 (71.9)	245 (71.0)	0.801
≥ 75 yr ─ no. (%)	111 (31.5)	106 (30.7)	0.818
Female sex ─ no. (%)	85 (24.2)	102 (29.6)	0.107
Race*─ no. (%)			0.256
White	331 (94.0)	331 (95.9)	
Black or African-American	2 (0.6)	0 (0.0)	
Asian	15 (4.3)	10 (2.9)	
American Indian or Alaska Native	0 (0.0)	0 (0.0)	
Other or unknown	4 (1.1)	4 (1.2)	
BMI†			
Median	28.4	29.1	**0.035**
Interquartile range	25.6 – 31.6	26.1 – 32.9	
Active smokers – no. (%)	30 (8.5)	24 (7.0)	0.480
Creatinine clearance ─ ml/min‡			
Mean	77.3 ± 31.4	82.3 ± 30.3	**0.037**
< 60 to ≥ 30 ml/min ─ no. (%)	90 (27.0)	75 (23.1)	0.280
<30 ml/min ─ no. (%)	4 (1.2)	2 (0.6)	0.686
$P2Y_{12}$ inhibitor at baseline – no. (%)			0.180
Clopidogrel	326 (92.6)	330 (95.7)	
Prasugrel	8 (2.3)	3 (0.9)	
Ticagrelor	18 (5.1)	12 (3.5)	
**Index Event**			
Type of Index Event – no. (%)			0.639
NSTEMI	62 (17.8)	66 (19.5)	
STEMI	50 (14.3)	38 (11.2)	
Unstable Angina	75 (21.5)	77 (22.8)	
Stable Angina	162 (46.4)	157 (46.5)	
Type of Stent – no. (%)			0.834
Drug-eluting stent	250 (71.6)	248 (72.3)	
Bare metal stent	90 (25.8)	84 (24.5)	
Drug-eluting and bare metal stents	9 (2.6)	11 (3.2)	
Urgency of Revascularization – no. (%)			0.879
Elective	200 (56.8)	198 (57.4)	
Urgent	152 (43.2)	147 (42.6)	
Type of Atrial Fibrillation ─ no. (%)			0.481

Characteristic	Group 2<br>Rivaroxaban + DAPT<br>(N = 352)	Group 3<br>VKA + DAPT<br>(N = 345)	p-value
Persistent	66 (18.8)	56 (16.3)	
Permanent	119 (33.8)	130 (37.8)	
Paroxysmal	167 (47.4)	158 (45.9)	
**Bleed Risk Scores**			
$CHADS_2$ risk of stroke – no. (%)			0.522
0	48 (13.6)	44 (12.8)	
1	119 (33.8)	119 (34.5)	
2	116 (33.0)	127 (36.8)	
3	64 (18.2)	48 (13.9)	
4	5 (1.4)	7 (2.0)	
5	0	0	
6	0	0	
CHA_2DS_2-VASc risk of stroke – no. (%)			0.035
0	6 (1.7)	4 (1.2)	
1	33 (9.4)	28 (8.1)	
2	56 (15.9)	44 (12.8)	
3	52 (14.8)	81 (23.5)	
4	78 (22.2)	80 (23.2)	
5	84 (23.9)	58 (16.8)	
6	31 (8.8)	41 (11.9)	
7	12 (3.4)	9 (2.6)	
HAS Bled Score – no. (%)			0.520
0	1 (0.3)	0 (0.0)	
1	28 (8.0)	18 (5.2)	
2	95 (27.0)	95 (27.5)	
3	149 (42.3)	150 (43.5)	
4	68 (19.3)	70 (20.3)	
5	11 (3.1)	10 (2.9)	
6	0 (0.0)	2 (0.6)	
**Comorbidities**			
Congestive heart failure	99 (28.1)	82 (23.8)	0.190
Hypertension	254 (72.2)	254 (73.6)	0.664
Diabetes mellitus	99 (28.1)	103 (29.9)	0.615
Hypercholesterolemia	148 (42.1)	141 (40.9)	0.753
Previous myocardial infarction	99 (28.1)	80 (23.2)	0.136
Peripheral vascular disease	23 (6.5)	16 (4.6)	0.276
Gastrointestinal bleeding	7 (2.0)	2 (0.6)	0.177
**Medications**			
Aspirin[¥]	351 (99.7)	344 (99.7)	>0.999
Beta-blocker	261 (74.2)	267 (77.4)	0.318
ACE inhibitor or ARB	250 (71.0)	266 (77.1)	0.067

Characteristic	Group 2<br>Rivaroxaban + DAPT<br>(N = 352)	Group 3<br>VKA + DAPT<br>(N = 345)	p-value
Statin	284 (80.7)	287 (83.2)	0.390
Proton pump inhibitor			0.792
Omeprazole or esomeprazole	39 (11.1)	38 (11.0)	
Other	97 (27.6)	103 (29.9)	

*Race was self-reported.
† Body mass index (BMI) is the weight (kg) divided by the square of the height (m).
‡ Creatinine clearance calculated using the Cockcroft-Gault equation.
¥ Aspirin use was calculated as administration of aspirin no more than 4 days after PCI procedure for index event.
Note: Plus-minus values are mean ± SD. ACE denotes angiotensin-converting enzyme, ACS acute coronary syndrome, ARB angiotensin-receptor blocker, BMI denotes body mass index, PCI percutaneous coronary intervention, NSTEMI non-ST-segment elevation myocardial infarction, STEMI ST-segment elevation myocardial infarction.
Note: Numbers based upon all randomized subjects.
Note: Pairwise comparisons were calculated using the chi-square test of independence for categorical variables, independent samples t-test for parametric continuous variables, and Wilcoxon rank sum test for non-parametric continuous variables.

Figure S1: Subgroup Analysis of All-Cause Death or First Recurrent Hospitalization
15 mg Rivaroxaban plus $P2Y_{12}$ Inhibitors vs. VKA plus DAPT

Subgroup	15 mg Riva plus $P2Y_{12}$	VKA plus DAPT	HR (95% CI)	P-Value[a]	P-Value[b]
Overall	228 / 696 (34.9)	261 / 697 (41.9)	0.79 (0.66 - 0.94)	0.008	
Sex					0.208
Male	154 / 517 (31.8)	188 / 513 (40.7)	0.73 (0.59 - 0.90)	0.004	
Female	74 / 179 (43.7)	73 / 184 (45.5)	0.94 (0.68 - 1.29)	0.693	
Age Group 1					0.173
< 75 years	122 / 445 (29.2)	168 / 473 (39.4)	0.70 (0.55 - 0.88)	0.002	
>= 75 years	106 / 251 (44.9)	93 / 224 (47.3)	0.90 (0.68 - 1.19)	0.451	
Age Group 2					0.777
< 65 years	42 / 184 (24.0)	50 / 179 (31.3)	0.74 (0.49 - 1.12)	0.155	
>= 65 years	186 / 512 (38.9)	211 / 518 (45.6)	0.80 (0.65 - 0.97)	0.024	
Race					0.112
White	212 / 650 (34.7)	248 / 655 (42.3)	0.76 (0.64 - 0.92)	0.004	
Black/ African American	1 / 7 (14.3)	0 / 1 (.)		0.706	
Asian	11 / 24 (48.4)	8 / 33 (28.3)	2.20 (0.88 - 5.46)	0.082	
Other	4 / 15 (29.1)	5 / 8 (70.8)	0.39 (0.10 - 1.47)	0.150	
Type of $P2Y_{12}$ Inhibitor					0.804
Clopidogrel	210 / 648 (34.3)	246 / 671 (41.1)	0.79 (0.66 - 0.95)	0.011	
Prasugrel	4 / 12 (41.7)	3 / 5 (60.0)	0.61 (0.14 - 2.72)	0.510	
Ticagrelor	14 / 36 (45.2)	12 / 21 (58.7)	0.65 (0.30 - 1.41)	0.275	
Type of Stent					0.584
Drug eluting stent	151 / 452 (35.7)	166 / 463 (40.4)	0.84 (0.68 - 1.05)	0.128	
Bare metal stent	73 / 230 (33.7)	90 / 221 (45.2)	0.69 (0.50 - 0.93)	0.016	
Both	4 / 14 (29.9)	4 / 11 (37.7)	0.79 (0.20 - 3.18)	0.743	
Region					0.281
North America	22 / 77 (29.2)	23 / 63 (42.7)	0.64 (0.35 - 1.14)	0.127	
East Europe	85 / 289 (30.7)	100 / 296 (38.2)	0.76 (0.57 - 1.02)	0.065	
West Europe	96 / 251 (42.4)	115 / 257 (49.5)	0.77 (0.59 - 1.01)	0.062	
Latin America	15 / 57 (27.8)	14 / 45 (34.5)	0.85 (0.41 - 1.76)	0.660	
Asia Pacific	10 / 22 (48.7)	9 / 36 (27.7)	2.09 (0.85 - 5.16)	0.100	

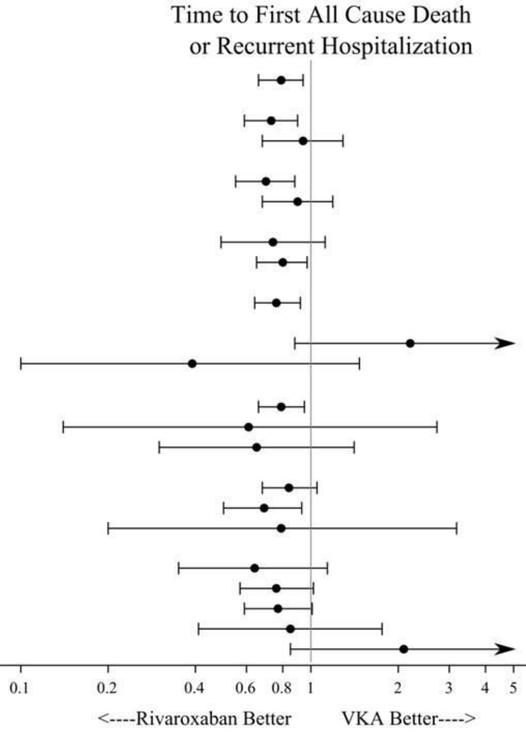

Note: Treatment-emergent period: period starting after the first study drug administration following randomization and ending 2 days after stop of study drug.
Note: KM Estimate represents rate of first re-hospitalization from treatment start date to 360 days of study direction.
Note: A subject could have more than one component event. n = number of subjects with events, N = number of subjects at risk.
Note: Hazard ratios as compared to the VKA group are based on the Cox proportional hazards model.
Note: Rehospitalizations do not include first index event hospitalization.
[a]Log-Rank p-values as compared to VKA group are based on the two-sided log rank test.
[b] P-Value for Interaction based on the Cox proportional Hazard joint test. References for joint test are as follow; Sex; male; Age Group 1: < 75 years; Age Group 2: < 65 years; Race: White; Type of $P2Y_{12}$ inhibitor: Clopidogrel; Type of Stent; Drug Eluting Stent; Region: North America.

Figure S2: Subgroup Analysis of All Cause Death or First Recurrent Hospitalization
2.5 mg Rivaroxaban plus DAPT vs. VKA plus DAPT

Subgroup	2.5 mg Riva plus DAPT	VKA plus DAPT	HR (95% CI)	P-Value[a]	P-Value[b]
Overall	213 / 706 (31.9)	261 / 697 (41.9)	0.75 (0.62 - 0.90)	0.002	
Sex					0.198
Male	167 / 533 (32.9)	188 / 513 (40.7)	0.80 (0.65 - 0.98)	0.033	
Female	46 / 173 (28.4)	73 / 184 (45.5)	0.60 (0.42 - 0.87)	0.007	
Age Group 1					0.423
< 75 years	123 / 461 (27.9)	168 / 473 (39.4)	0.70 (0.55 - 0.88)	0.002	
>= 75 years	90 / 245 (39.6)	93 / 224 (47.3)	0.81 (0.61 - 1.09)	0.159	
Age Group 2					0.427
< 65 years	48 / 191 (26.0)	50 / 179 (31.3)	0.86 (0.58 - 1.27)	0.444	
>= 65 years	165 / 515 (34.1)	211 / 518 (45.6)	0.72 (0.59 - 0.88)	0.002	
Race					0.671
White	203 / 668 (32.1)	248 / 655 (42.3)	0.74 (0.62 - 0.90)	0.002	
Black/ African American	0 / 3 (.)	0 / 1 (.)			
Asian	8 / 28 (28.6)	8 / 33 (28.3)	1.11 (0.41 - 2.95)	0.840	
Other	2 / 7 (28.6)	5 / 8 (70.8)	0.36 (0.07 - 1.90)	0.212	
Intended DAPT Duration					0.222
1 Month	48 / 108 (45.1)	53 / 113 (51.2)	0.92 (0.62 - 1.35)	0.667	
6 Months	80 / 248 (34.8)	92 / 243 (42.7)	0.84 (0.62 - 1.13)	0.243	
12 Months	85 / 350 (25.6)	116 / 341 (38.2)	0.63 (0.47 - 0.83)	0.001	
Type of $P2Y_{12}$ Inhibitor					0.414
Clopidogrel	197 / 662 (31.5)	246 / 671 (41.1)	0.76 (0.63 - 0.91)	0.003	
Prasugrel	2 / 11 (18.2)	3 / 5 (60.0)	0.22 (0.04 - 1.31)	0.067	
Ticagrelor	14 / 33 (43.4)	12 / 21 (58.7)	0.66 (0.31 - 1.44)	0.296	
Type of Stent					0.368
Drug eluting stent	145 / 468 (32.8)	166 / 463 (40.4)	0.81 (0.64 - 1.01)	0.056	
Bare metal stent	65 / 220 (31.1)	90 / 221 (45.2)	0.67 (0.49 - 0.92)	0.012	
Both	2 / 14 (14.3)	4 / 11 (37.7)	0.35 (0.06 - 1.89)	0.200	
Region					0.137
North America	19 / 73 (27.7)	23 / 63 (42.7)	0.62 (0.34 - 1.12)	0.110	
East Europe	69 / 307 (23.7)	100 / 296 (38.2)	0.60 (0.44 - 0.82)	0.001	
West Europe	95 / 244 (41.9)	115 / 257 (49.5)	0.82 (0.63 - 1.08)	0.162	
Latin America	20 / 51 (40.2)	14 / 45 (34.5)	1.35 (0.68 - 2.67)	0.386	
Asia Pacific	10 / 31 (32.3)	9 / 36 (27.7)	1.28 (0.52 - 3.16)	0.586	

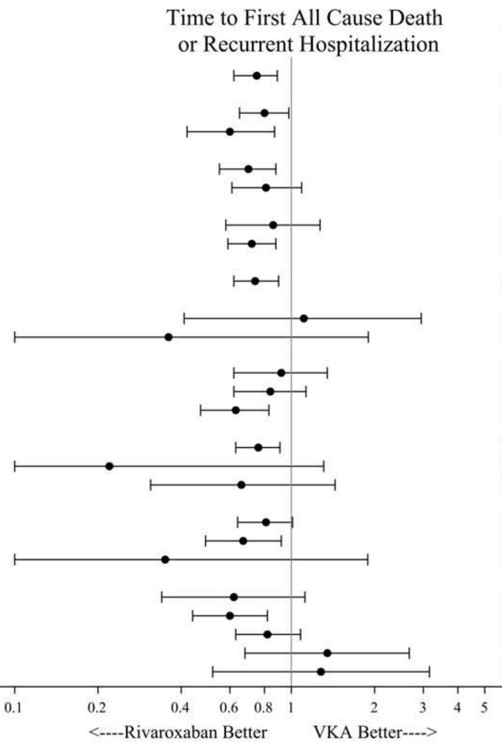

Note: Treatment-emergent period: period starting after the first study drug administration following randomization and ending 2 days after stop of study drug.
Note: KM Estimate represents rate of first re-hospitalization from treatment start date to 360 days of study direction.
Note: A subject could have more than one component event. n = number of subjects with events, N = number of subjects at risk.
Note: Hazard ratios as compared to the VKA group are based on the Cox proportional hazards model.
Note: Rehospitalizations do not include first index event hospitalization.
[a]Log-Rank p-values as compared to VKA group are based on the two-sided log rank test.
[b] P-Value for Interaction based on the Cox proportional Hazard joint test. References for joint test are as follow; Sex; male; Age Group 1: < 75 years; Age Group 2: < 65 years; Race: White; Intended DAPT Duration: 1 Month; Type of $P2Y_{12}$ inhibitor: Clopidogrel; Type of Stent; Drug Eluting Stent; Region: North America.

Figure S3: Subgroup Analysis of First Recurrent Hospitalization
15 mg Rivaroxaban plus $P2Y_{12}$ Inhibitors vs. VKA plus DAPT

Subgroup	15 mg Riva plus $P2Y_{12}$	VKA plus DAPT	HR (95% CI)	P-Value[a]	P-Value[b]
Overall	221 / 696 (34.1)	257 / 697 (41.5)	0.77 (0.65- 0.92)	0.005	
Sex					0.176
Male	149 / 517 (31.0)	186 / 513 (40.4)	0.71 (0.57 - 0.88)	0.002	
Female	72 / 179 (42.9)	71 / 184 (44.8)	0.94 (0.67 - 1.30)	0.691	
Age Group 1					0.127
< 75 years	116 / 445 (28.0)	165 / 473 (39.0)	0.67 (0.53 - 0.85)	0.001	
>= 75 years	105 / 251 (44.6)	92 / 224 (47.1)	0.90 (0.68 - 1.19)	0.454	
Age Group 2					0.563
< 65 years	39 / 184 (22.5)	50 / 179 (31.3)	0.69 (0.45 - 1.05)	0.082	
>= 65 years	182 / 512 (38.3)	207 / 518 (45.1)	0.79 (0.65 - 0.97)	0.023	
Race					0.107
White	205 / 650 (33.9)	244 / 655 (41.9)	0.75 (0.62 - 0.90)	0.003	
Black/ African American	1 / 7 (14.3)	0 / 1 (.)		0.706	
Asian	11 / 24 (48.4)	8 / 33 (28.3)	2.20 (0.88 - 5.46)	0.082	
Other	4 / 15 (29.1)	5 / 8 (70.8)	0.39 (0.10 - 1.47)	0.150	
Type of $P2Y_{12}$ Inhibitor					0.828
Clopidogrel	203 / 648 (33.4)	242 / 671 (40.7)	0.77 (0.64 - 0.93)	0.007	
Prasugrel	4 / 12 (41.7)	3 / 5 (60.0)	0.61 (0.14 - 2.72)	0.510	
Ticagrelor	14 / 36 (45.2)	12 / 21 (58.7)	0.65 (0.30 - 1.41)	0.275	
Type of Stent					0.350
Drug eluting stent	150 / 452 (35.5)	163 / 463 (40.0)	0.85 (0.68 - 1.06)	0.157	
Bare metal stent	68 / 230 (31.9)	89 / 221 (44.8)	0.65 (0.47 - 0.88)	0.006	
Both	3 / 14 (24.0)	4 / 11 (37.7)	0.59 (0.13 - 2.64)	0.484	
Region					0.266
North America	22 / 77 (29.2)	23 / 63 (42.7)	0.64 (0.35 - 1.14)	0.127	
East Europe	83 / 289 (30.1)	98 / 296 (37.6)	0.76 (0.57 - 1.02)	0.064	
West Europe	92 / 251 (41.3)	114 / 257 (49.3)	0.75 (0.57 - 0.98)	0.036	
Latin America	14 / 57 (26.1)	13 / 45 (33.0)	0.85 (0.40 - 1.81)	0.674	
Asia Pacific	10 / 22 (48.7)	9 / 36 (27.7)	2.09 (0.85 - 5.16)	0.100	

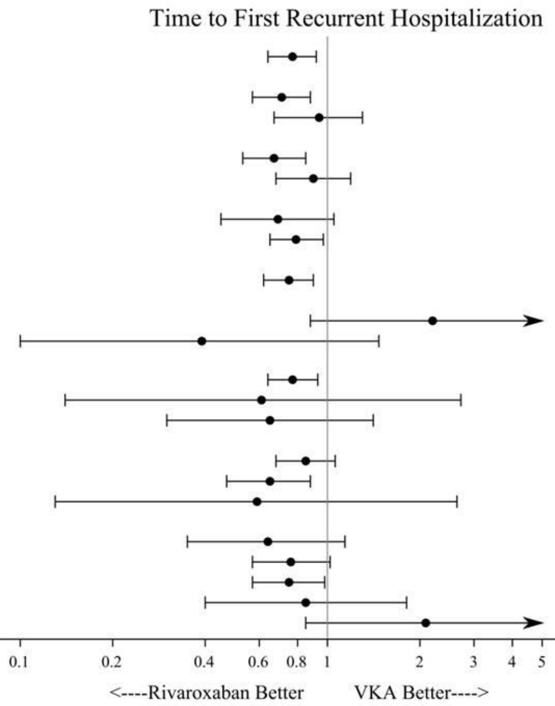

Note: Treatment-emergent period: period starting after the first study drug administration following randomization and ending 2 days after stop of study drug.
Note: KM Estimate represents rate of first re-hospitalization from treatment start date to 360 days of study direction.
Note: A subject could have more than one component event. n = number of subjects with events, N = number of subjects at risk.
Note: Hazard ratios as compared to the VKA group are based on the Cox proportional hazards model.
Note: Rehospitalizations do not include first index event hospitalization.

[a]Log-Rank p-values as compared to VKA group are based on the two-sided log rank test.

[b] P-Value for Interaction based on the Cox proportional Hazard joint test. References for joint test are as follow; Sex; male; Age Group 1: < 75 years; Age Group 2: < 65 years; Race: White; Type of $P2Y_{12}$ inhibitor: Clopidogrel; Type of Stent; Drug Eluting Stent; Region: North America.

Figure S4: Subgroup Analysis of First Recurrent Hospitalization
2.5 mg Rivaroxaban plus DAPT vs. VKA plus DAPT

Subgroup	2.5 mg Riva plus DAPT	VKA plus DAPT	HR (95% CI)	P-Value[a]	P-Value[b]
Overall	207 / 706 (31.2)	257 / 697 (41.5)	0.74 (0.61 - 0.88)	0.001	
Sex					0.201
Male	163 / 533 (32.3)	186 / 513 (40.4)	0.79 (0.64 - 0.97)	0.025	
Female	44 / 173 (27.3)	71 / 184 (44.8)	0.59 (0.41 - 0.86)	0.006	
Age Group 1					0.363
< 75 years	118 / 461 (26.9)	165 / 473 (39.0)	0.68 (0.54 - 0.86)	0.001	
>= 75 years	89 / 245 (39.3)	92 / 224 (47.1)	0.81 (0.61 - 1.09)	0.158	
Age Group 2					0.616
< 65 years	45 / 191 (24.5)	50 / 179 (31.3)	0.81 (0.54 - 1.20)	0.289	
>= 65 years	162 / 515 (33.7)	207 / 518 (45.1)	0.72 (0.59 - 0.88)	0.002	
Race					0.666
White	197 / 668 (31.4)	244 / 655 (41.9)	0.73 (0.61 - 0.89)	0.001	
Black/ African American	0 / 3 (.)	0 / 1 (.)			
Asian	8 / 28 (28.6)	8 / 33 (28.3)	1.11 (0.41 - 2.95)	0.840	
Other	2 / 7 (28.6)	5 / 8 (70.8)	0.36 (0.07 - 1.90)	0.212	
Intended DAPT Duration					0.205
1 Month	48 / 108 (45.1)	53 / 113 (51.2)	0.92 (0.62 - 1.35)	0.667	
6 Months	77 / 248 (33.8)	90 / 243 (42.2)	0.82 (0.61 - 1.12)	0.209	
12 Months	82 / 350 (24.8)	114 / 341 (37.7)	0.62 (0.46 - 0.82)	0.001	
Type of $P2Y_{12}$ Inhibitor					0.261
Clopidogrel	192 / 662 (30.9)	242 / 671 (40.7)	0.75 (0.62 - 0.90)	0.002	
Prasugrel	1 / 11 (9.1)	3 / 5 (60.0)	0.11 (0.01 - 1.06)	0.021	
Ticagrelor	14 / 33 (43.4)	12 / 21 (58.7)	0.66 (0.31 - 1.44)	0.296	
Type of Stent					0.282
Drug eluting stent	143 / 468 (32.4)	163 / 463 (40.0)	0.81 (0.65 - 1.01)	0.063	
Bare metal stent	61 / 220 (29.6)	89 / 221 (44.8)	0.63 (0.46 - 0.88)	0.005	
Both	2 / 14 (14.3)	4 / 11 (37.7)	0.35 (0.06 - 1.89)	0.200	
Region					0.076
North America	18 / 73 (26.4)	23 / 63 (42.7)	0.59 (0.32 - 1.08)	0.081	
East Europe	65 / 307 (22.5)	98 / 296 (37.6)	0.58 (0.42 - 0.79)	0.001	
West Europe	94 / 244 (41.6)	114 / 257 (49.3)	0.82 (0.63 - 1.08)	0.159	
Latin America	20 / 51 (40.2)	13 / 45 (33.0)	1.46 (0.72 - 2.93)	0.287	
Asia Pacific	10 / 31 (32.3)	9 / 36 (27.7)	1.28 (0.52 - 3.16)	0.586	

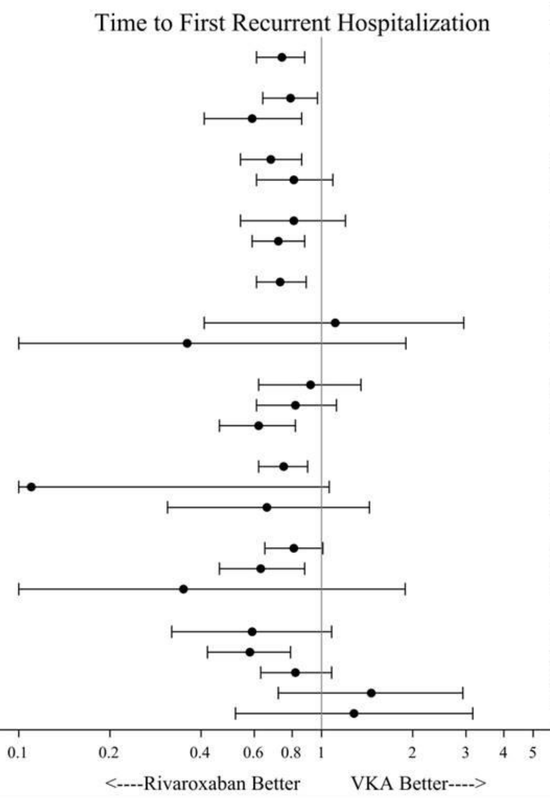

Note: Treatment-emergent period: period starting after the first study drug administration following randomization and ending 2 days after stop of study drug.
Note: KM Estimate represents rate of first re-hospitalization from treatment start date to 360 days of study direction.
Note: A subject could have more than one component event. n = number of subjects with events, N = number of subjects at risk.
Note: Hazard ratios as compared to the VKA group are based on the Cox proportional hazards model.
Note: Rehospitalizations do not include first index event hospitalization.

[a]Log-Rank p-values as compared to VKA group are based on the two-sided log rank test.
[b] P-Value for Interaction based on the Cox proportional Hazard joint test. References for joint test are as follow; Sex; male; Age Group 1: < 75 years; Age Group 2: < 65 years; Race: White; Intended DAPT Duration: 1 Month; Type of $P2Y_{12}$ inhibitor: Clopidogrel; Type of Stent; Drug Eluting Stent; Region: North America.
